# Cold comfort for change: Stream mats as biological indicators of ecosystem processes in the McMurdo Dry Valleys, Antarctica

**DOI:** 10.1111/jpy.70142

**Published:** 2026-02-28

**Authors:** Tyler J. Kohler, Ian Hawes, Adrian Howkins, Lydia H. Zeglin, Mike N. Gooseff, Diane M. McKnight

**Affiliations:** ^1^ Department of Ecology, Faculty of Science Charles University Prague Czech Republic; ^2^ Coastal Marine Group University of Waikato Tauranga New Zealand; ^3^ Department of Historical Studies, School of Humanities University of Bristol Bristol UK; ^4^ Division of Biology Kansas State University Manhattan Kansas USA; ^5^ Institute of Arctic and Alpine Research University of Colorado Boulder Colorado USA

**Keywords:** Antarctic diatom, cyanobacteria, freshwater ecology, glacier‐fed stream, McMurdo Dry Valleys, microbial mat

## Abstract

Glacier‐fed streams (GFSs) make ideal systems for studying climate‐related changes. Some of the best‐studied GFSs are found in the McMurdo Dry Valleys (MDVs) of Antarctica, one of the Earth's coldest and driest deserts. Despite their harsh and isolated nature, MDV GFSs represent an oasis of life in a landscape visually devoid of it, with biomass dominated by photosynthetic microorganisms (including chlorophytes, cyanobacteria, and diatoms) and manifesting as benthic “mats.” Mats form the basis of MDV GFS ecosystems, drive biogeochemical cycles, and harbor high proportions of the regional biodiversity. Furthermore, the biomass and composition of these mats respond to environmental fluctuations, making them ideal bioindicators for ecological monitoring. In this review, we have (1) distinguished the three major photosynthetic mat types by their taxonomic structure, habitat use, and elemental composition; (2) demonstrated how mat type distribution, coverage, and biomass are dictated by a combination of geomorphology, suspended sediment loads, and hydrology, among other factors; (3) introduced MDV diatoms as model organisms for investigating mat community assembly; and (4) speculated on how the biomass, community structure, and functional process rates of different mat types will change in a warmer and more connected world. Synthesizing this information, we suggest future opportunities for research, with the most promising avenues centering upon questions, methodologies, and scales that would have been inconceivable for the Heroic Age explorers that discovered them, ranging from studies of gene expression to cataloging changes in mat abundance by satellite.

**Abbreviations**
AFDM: ash‐free dry mass
ASV: amplicon sequence variant
GFS: glacier‐fed stream
LiDAR: light detection and ranging
MCMLTER: McMurdo Long‐Term Ecological Research
MDV: McMurdo Dry Valley
OTU: operational taxonomic unit
UV: ultra‐violet

## INTRODUCTION

### Glacier‐fed streams of the McMurdo Dry Valleys

Global climate changes continue to intensify and greatly influence Earth's hydrological cycles (International Panel on Climate Change, 2019). Epitomizing these global changes are *glacier‐fed streams*. Glacier‐fed streams (GFSs, also known as “kryal” streams; Ward, 1994) are located in alpine and polar regions worldwide and collect the liquid remains of glaciers into channels, transporting them to lower elevations. The retreat of glaciers globally has been well documented, and changes to glacier melt rates have fairly well‐known physical and chemical consequences for GFSs, which include altering the flux of water, suspended materials, and solutes to downstream ecosystems. In contrast, the biological responses to glacier recession remain poorly understood, and accordingly, the last decade has seen research attention on GFSs refocus from shifts in their specific environmental characteristics to how these changes drive community structure and function (e.g. Hotaling et al., 2017).

The environmental template of GFSs is unique among stream types (Uehlinger et al., 2010; Ward, 1994). Being a product of glacial melt, GFS discharge is intimately related to the energy balance of glacial surfaces, which is in turn influenced by ambient temperature and solar radiation. As a result, most discharge is limited to summer months, with daily hydrological peaks coinciding with afternoon radiative peaks, and winters prone to drying and/or complete freezing. Longitudinal patterns are also present, with streamwater temperatures near freezing at glacial termini and slowly increasing downstream as radiative energy accumulates. The alpine setting of most GFSs also creates steep slopes, resulting in turbulent flows that mobilize benthic sediments, scour loosely attached biota, and promote sunlight‐attenuating turbidity. Finally, due to poorly vegetated catchments at high latitudes/elevations, most GFSs have low allochthonous organic matter inputs, with local metabolisms driven by in‐stream productivity or recently exposed mineral surfaces. These conditions, together with their truncated upper trophic levels, give GFSs the reputation of being some of Earth's harshest freshwater ecosystems.

**FIGURE 1:**
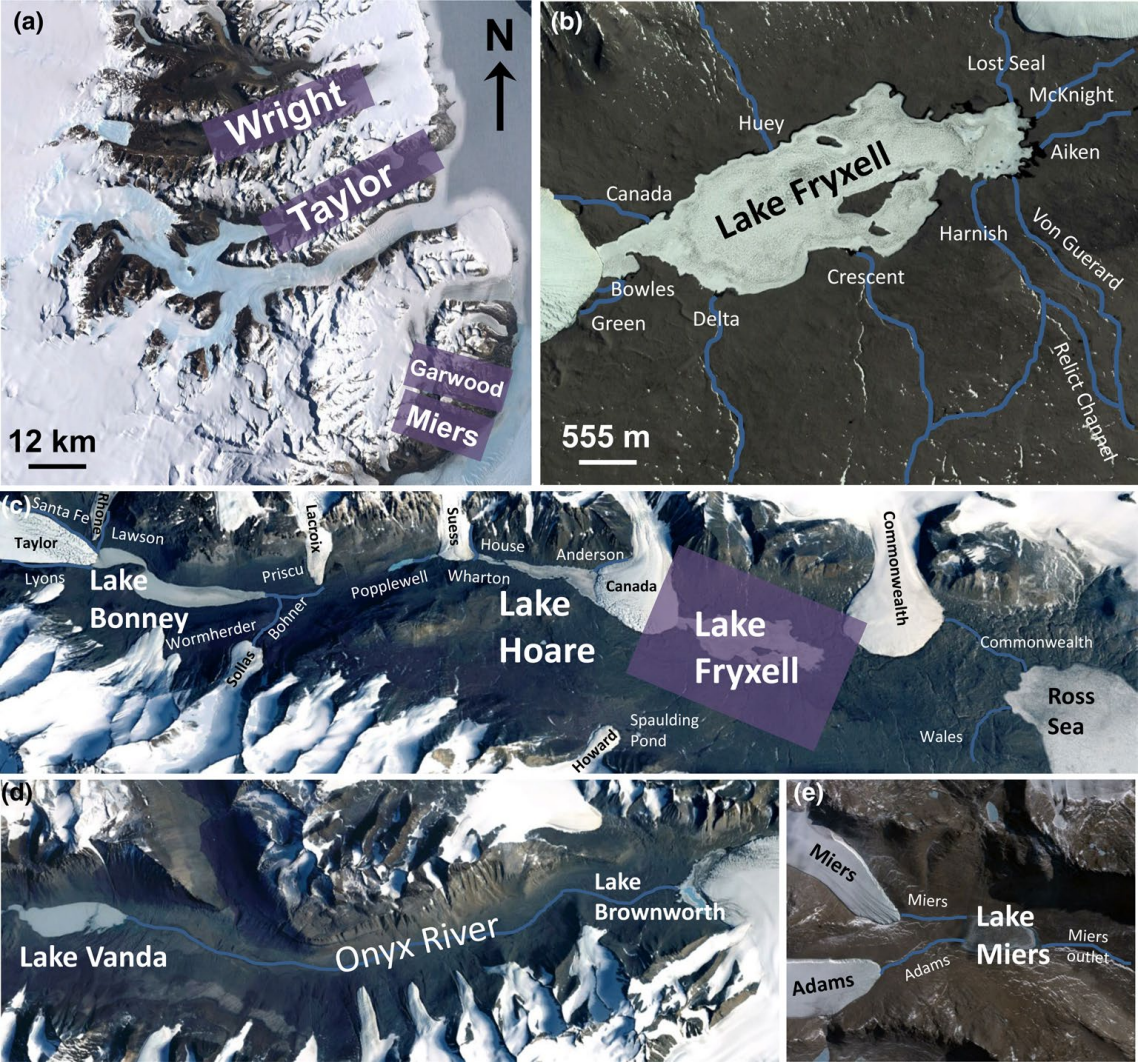
The McMurdo Dry Valleys are the largest continuous ice‐free region of Antarctica. Shown here is (a) a satellite image of the entire McMurdo Dry Valley region, directly west of Ross Island, with the positions of Wright, Taylor, Garwood, and Miers valleys highlighted; (b) a detail of the Lake Fryxell basin, with major streams identified; (c) Taylor Valley, with major lakes, glaciers, and streams labeled (see panel B for names of streams draining into Lake Fryxell); (d) a detail of Wright Valley, with the two major lakes and the Onyx River identified; and (e) a detail of Miers Valley, with major features labeled. Background imagery courtesy USGS (a), Maxar Technologies (b), and Landsat (c–e), all acquired via GoogleEarth.

Within this world of “extreme streams,” the GFSs in the McMurdo Dry Valleys (MDVs) of Antarctica may be some of the most extreme of all given their geographical position. Nonetheless, due to the past efforts by the New Zealand and United States Antarctic programs, they are also some of the best studied. Situated in South Victoria Land at the foot of the Transantarctic Mountains (76.830–78.800° S, 160.800–165.800° E; Figure 1a), the MDVs make up Antarctica's largest individual ice‐free area (encompassing >4500 km^2^; Levy, 2013) as well as one of its oldest areas, with some areas being ice‐free for millions of years (Fountain et al., 1999). Here, the average temperature is −19°C, although lows can approach −60°C during the 24‐h darkness of winter, and highs can reach 10°C during the 24‐h sunlight of summer (Obryk et al., 2020). Coupled with maximum wind speeds of ~40 m · s^−1^ and <10 cm of annual precipitation, these conditions make the MDV one of the coldest, windiest, and driest places on Earth. This harshness is reflected in the barren landscape, where macroscopic life is limited to the occasional moss cushion or lichen‐encrusted rock.

Yet, the MDV GFSs serve as oases for life within this seemingly empty world by harboring high abundances of vividly colored mats upon their sediment surfaces. Being perennial, these mats create hotspots of both diversity and organic matter, and their redistribution by wind and water aids in facilitating life in a land where there is relatively little of everything (Nkem et al., 2006; Wharton Jr et al., 1983). Within GFSs, mats intercept, assimilate, and transform organic matter and nutrients en route to their outlet lakes (Gooseff et al., 2004), effectively dictating how much and in what form reactive solutes reach their final destination (Aiken et al., 1996; Dowling et al., 2014; Foreman et al., 2004; Green et al., 1988; Howard‐Williams et al., 1997). Thus, stream mats serve as biogeochemical liaisons among MDV glaciers, soils, and closed basin lakes, and understanding their ecology is, therefore, fundamental to understanding the MDV ecosystem as a whole.

Like Earth's other alpine and polar regions, the MDVs are changing (Fountain et al., 2014). Because of their local ecological importance and responsiveness to the external environment, MDV stream mats have the excellent potential to serve as bioindicators, tracking and documenting changes in real time. As with all bioindicators, however, their usefulness is directly related to our knowledge of their ecology, which dictates the scope of questions that can be asked of them. In this review, we have synthesized the last several decades of MDV GFS mat research, beginning with a short history of their inquiry and, thereafter, focusing on their diversity, composition, and ecological controls. Afterward, we have discussed how the diatoms, which live within stream mats, are an ideal model group for studying how these unique communities are assembled. Finally, we have discussed the future trajectory of the different mats and their constituent parts and suggested some of the most promising avenues for future research.

### A short history

Human history in the MDV is remarkably recent, beginning 18 December 1903 during Robert Falcon Scott's *Discovery* expedition (1901–1904). During his team's return from the polar plateau, he came across the upper part of Taylor Valley, about which he wrote, “It is certainly a valley of the dead; even the great glacier which once pushed through it has withered away” (Scott, 1905, vol. 2, p. 217). The next documented visit to the area occurred a few years later during Shackleton's *Nimrod* expedition (1907–1909), with Raymond Priestly leading a small party that spent a couple of days in the lower part of the ice‐free valley (Murray, 1910). Then, in February 1911, an expedition led by Griffith Taylor set out to explore the valley during Scott's *Terra Nova* expedition (1910–1913). He later wrote: “It was warm weather most of the time we spent in Dry Valley—rising sometimes above freezing‐point—and everywhere streams were tinkling among the black boulders, so much so that this valley, in spite of its name, was certainly the wettest area I saw in Antarctica!” (Taylor, 1913, vol. 2, p. 192).

Taylor, whom both the valley and its main glacier would later be named after (Figure 1a,c), was arguably the first to appreciate the role of liquid water in forming the MDV landscape as well the first to take a stream width and depth measurement (Howkins, 2016). Although he did not mention the stream mats specifically, he did note that a “large laminae of dull green algae covered the bottom of the lake,” which he named “Lake Bonney” (Taylor, 1916, p. 136). Nonetheless, mats were sampled from other places and habitats during the Heroic Age and returned to the old world with the explorers, where they were painstakingly processed to provide the first published observations of chlorophytes, cyanobacteria, and diatoms from the McMurdo Sound Region. As a result, a report corresponded with each of the *Discovery* (Fritsch, 1912), *Nimrod* (West & West, 1911), and *Terra Nova* (Fritsch, 1917) expeditions, teaching us much about the local flora and, indeed, ultimately becoming the phycological foundation for the entire region. Yet, it would be nearly a half‐century later before the MDV stream mats, specifically, would get their notice.

Interest in Antarctica was rekindled following World War II. Planes from the United States flew over and photographed portions of the MDV during Operation Highjump (1946–1947), and the MDVs were further explored on the ground during “Operation Deep Freeze” (1955) and the International Polar Year (1957–1958), providing a much better idea of the region's geographical scale. As it was part of their national claim, New Zealand led research efforts in the MDV from the late 1960s, which commenced with the opening of the Lake Vanda Station in Wright Valley (Figure 1a,d) in 1968–1969 (Howkins et al., 2021). There, researchers initiated new investigations into MDV geology, glaciology, and meteorology as well as some of the first work on streams. Notably, this work included the first discharge measurements of the Onyx River, the longest river in Antarctica (Castendyk et al., 2016; Chinn, 1993; Chinn & Mason, 2016). For almost 25 years, the “Asgard Rangers” kept hydrological measurements on Wright Valley as well as a subset on Taylor and Miers valleys (Figure 1a,e).

It was during this time that the first observations of stream life were made, with the earliest (to our knowledge) published by Seaburg et al. (1979) who reported on samples from the Miers and Bonney basins. Shortly thereafter, Broady (1982) reported on algae collected from Canada (then called “Fryxell”) Stream. Reinforcing the novelty of these systems at the time, Broady (1982) stated, “The lack of data on stream algae is noted as a significant gap in our knowledge of Antarctic algae” (p. 331), and “In view of the considerable number of summer meltwater streams in the ice‐free areas, most supporting rich algal growths, it is suggested that their biology deserves more detailed investigation” (p. 347).

Following these two primarily taxonomic surveys came much of the “classic” MDV stream ecology work, investigating everything from organismal physiology to nitrogen biogeochemistry (e.g., Downes et al., 1986; Hawes et al., 1992; Howard‐Williams et al., 1986, 1989; Vincent, Castenholz, et al., 1993; Vincent, Downes, et al., 1993; Vincent & Howard‐Williams, 1986, 1989). However, Vanda Station would be dismantled in the early 1990s (Hawes et al., 2025; Howkins et al., 2021), marking a transition in both the New Zealand Antarctic Program and MDV stream ecology. Concomitantly, United States researchers had been working on the lakes in Taylor Valley to the south. Inspired by the work in Wright Valley and with their own early limnological results in hand, the first proposal for a McMurdo Long‐Term Ecological Research (MCMLTER) program was submitted, with the funded project taking over MDV hydrological monitoring in the 1993–1994 summer. This program has continued to date and, with it, a “second generation” of MDV stream ecology work.

Importantly, researchers from both the New Zealand and United States programs realized that the lack of terrestrial vegetation, meaningful grazing activity, and anthropogenic influences, coupled with constrained water and nutrient sources, substantially simplified controlled investigations. Thus, MDV GFSs are one of the world's best places to study fundamental processes in stream ecology, spanning hydrology, community assembly, and biogeochemistry. For example, MDV GFSs and their inhabitants can be used as models for understanding biological responses to intra‐ and interannual flow extremes, with implications for the world's many other intermittent streams (Messager et al., 2021), as well as for identifying controls on nuisance (Cullis et al., 2012) or toxin‐producing (McAllister et al., 2016) mat biomass. Furthermore, MDV GFSs can be used as analogs for places inaccessible through space and time, such as Martian sediments (Bishop et al., 2001; Doran et al., 1998), the ancient Earth (Vincent, 2000; Vincent & Howard‐Williams, 2000), and even future space flights (Matula et al., 2021). Finally, given summer temperatures that effectively straddle the freezing/melting point of water, the balance of which dictates the presence and magnitude of stream discharge, stream mats make ideal biological indicators for the resilience of continental Antarctic ecosystems to climate change.

## STREAM MATS

### Etymology

Microbial mats are thick biofilms hosting eukaryotes, bacteria, archaea, and viruses within their matrix. They are observed worldwide, although most notably in chemically and thermally stressed environments with low grazing pressures, including arid deserts, hypersaline environments, and polar freshwaters (Paerl et al., 2000; Stal, 2000). In GFSs, microbial mats (hereafter mats) dominated by photoautotrophic microbes (e.g., cyanobacteria, chlorophytes) often develop as a response to the unique environmental template. Specifically, GFSs are highly intermittent, and mats must generally tolerate winters in either a frozen or freeze‐dried state. In summer, glacial melt leads to high and variable flows (promoting scour), and mats can be exposed to high levels of radiation. Finally, while streamwater temperatures and nutrient concentrations are generally low, suspended sediment concentrations are often high due to glacier weathering and unconsolidated substrata. Collectively, these characteristics represent considerable challenges for the presence and long‐term persistence of stream life.

Yet, mats can thrive despite these high‐stress conditions and are readily observed within and along the margins of alpine (Miller & Lane, 2019; Roncoroni et al., 2019), Arctic (Elster et al., 1997; Komárek et al., 2012; Palinska et al., 2017), and Maritime Antarctic (Almela et al., 2019; Hawes, 1989, 1993; Hawes & Brazier, 1991; Komárek & Elster, 2008) GFSs, with MDV streams being no exception (Broady, 1982; Howard‐Williams et al., 1986; McKnight et al., 1998). Across all of these environments, a high degree of consistency has been observed in terms of the communities that ultimately assemble, both within the stream and its subhabitats (e.g., Elster et al., 1997; Hawes & Brazier, 1991; Komárek et al., 2012; Komárek & Elster, 2008). For example, communities dominated by filamentous cyanobacteria tend to form encrusting, persistent growths on stream bottom sediments. Meanwhile, mucilaginous colonies of *Nostoc* form along stream margins and seepages, whereas tufts and streamers of green and yellow‐green algae are common annual developments filling the interstitial spaces of stones.

In the MDVs, resident stream mats were initially categorized according to their most abundant constituents, with communities dominated by *Phormidium* and *Nostoc* termed *Phormidium* mats and *Nostoc* mats, respectively (e.g., Vincent & Howard‐Williams, 1986, 1989). As elsewhere, this classification of MDV mats was shown to correspond to different stream subhabitats, with *Nostoc* mats consistently observed at stream margins and *Phormidium* mats occupying the main channel. Later, a color‐based system for mat classification was introduced by Alger (1997) and McKnight et al. (1998), originating as a solution to deal with the greater taxonomic diversity that emerged within some mat types as the number of observations increased. Although we recognize that this system is imperfect, it does have research history and ecological relevance and promotes consistency and efficiency during field campaigns. Hence, we have herein delineated three major MDV mat types based on their color, which we have shown corresponds to predictable differences in community composition, streambed habitat, and elemental/isotopic signatures.

### Mat types

#### Orange mats

**FIGURE 2:**
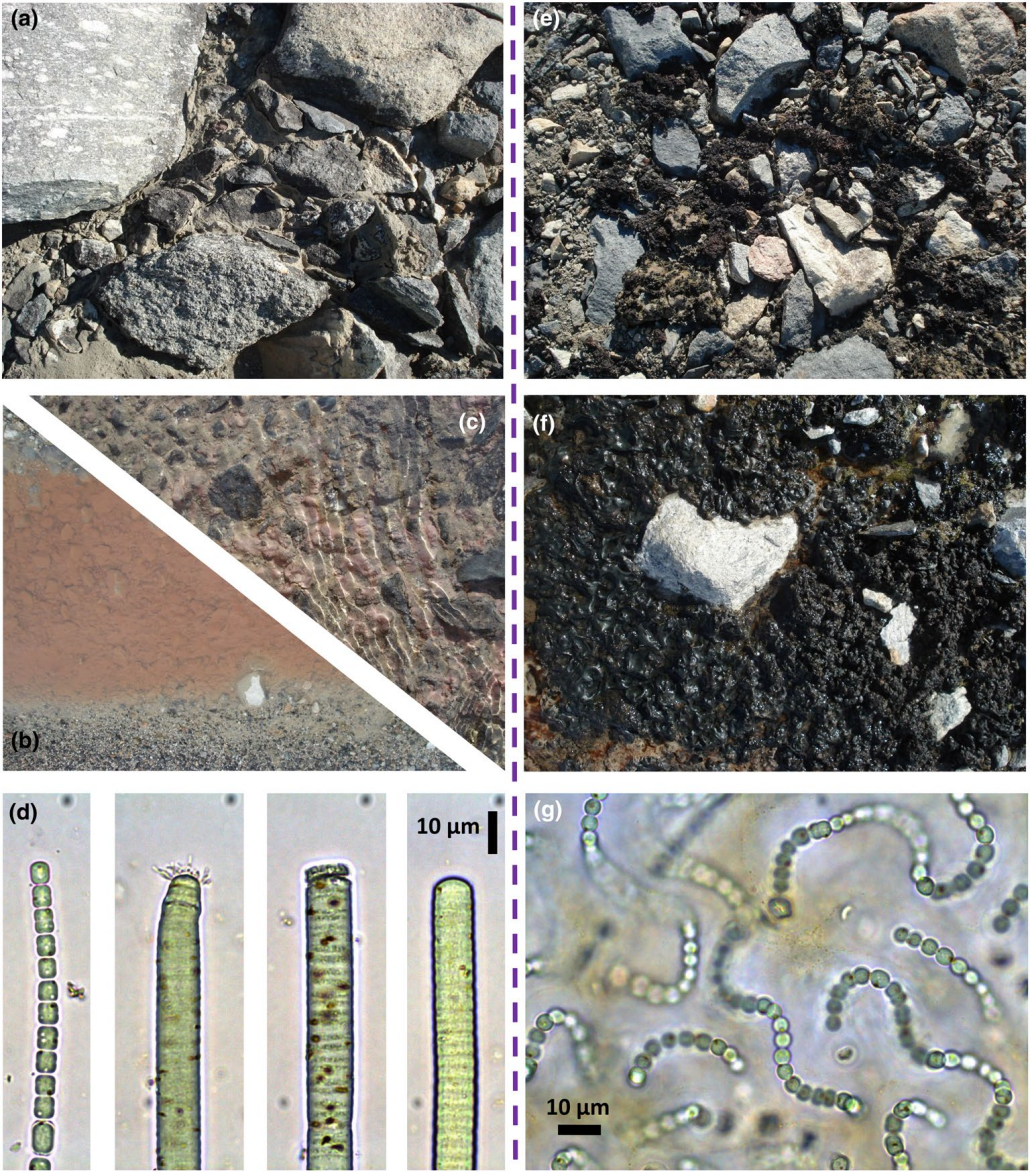
Orange and black mats make up most of the visible biomass in McMurdo Dry Valley streams, and examples of each from the field and under the microscope are shown here. For orange mats (left column), pictured are (a) dried orange mats from Canada Stream, (b) hydrated orange mats from Priscu Stream, (c) hydrated “red” mats from Canada Stream, and (d) four filamentous cyanobacterial trichomes composing an orange mat from Green Creek (the scale bar in the last subpanel applies to all subpanels of d). For black mats (right column), pictured are (e) dried black mats from Canada Stream, (f) hydrated black mats from Canada Stream, and (g) a microscopic view of a *Nostoc* colony from Green Creek. All images by T.J. Kohler.

Orange mats occupy the central channel of streambeds and, like their name suggests, are often vibrant orange in color (Figure 2a,b). The “foundation” of orange mats is formed primarily by filamentous cyanobacteria (Figure 2d), with the most widely reported being species of the family Oscillatoriaceae (e.g. Alger, 1997; McKnight et al., 1998; Vincent, Castenholz, et al., 1993; Vincent, Downes, et al., 1993). However, it is challenging to identify filamentous cyanobacteria via microscopy due to overlapping descriptions, frequent updates to taxonomy, and within‐taxon variability. This situation is especially true for groups with fine trichomes (e.g. *Leptolyngbya*, *Pseudanabaena*, and *Phormidesmis*), within which taxa have few visible identifiable characteristics (Komárek, 2007; Komárek et al., 2009). Past investigators have coped with these challenges by focusing on trichome morphometrics (Broady & Kibblewhite, 1991), by creating different morphotype categories (Alger, 1997; McKnight et al., 1998), or by analyzing these groups to the genus level only (Broady, 1996; Kohler, Chatfield, et al., 2015).

Happily, environmental sequencing methods can potentially improve upon this frustrating situation (Dvořák et al., 2015). Of the few molecular‐based studies available, researchers have generally observed *Phormidium* to have high relative abundances in orange mats (Kohler et al., 2016; Stanish, O'Neill, et al., 2013), aligning with past morphological observations. However, other filamentous cyanobacteria, such as *Tychonema, Kamtonema*, and *Willmottia*, have also been observed at high relative abundances (Zoumplis et al., 2023), which potentially highlights some of the challenges that come along with using molecular methods, including quantifying prokaryote diversity (e.g., prokaryote species definitions, primer selection, operational taxonomic units vs. amplicon sequence variants) and assigning names to a given sequence (e.g., which databases are used for identification and past taxonomic revisions—for example, *Phormidium autumnale* has been revised to *Microcoleus autumnalis*; Strunecký et al., 2013). Nonetheless, the relative speed and volume of data that can be acquired, coupled with the potential for more objective and replicable results among analysts (e.g., especially important for questions of biogeography), make community profiling by DNA sequencing a promising avenue for future MDV research.

Irrespective of how orange mat diversity is characterized, previous works have agreed that the taxonomic composition of MDV orange mats varies widely, both across streams and within stream reaches. Indeed, orange mats have been long appreciated to be “hotspots” in terms of their diversity. Fritsch (1917, p. 1) noted that “…the Cyanophyceous flora, and particularly that occurring on the Phormidium‐sheets, is by no means exhausted” and even suggested, “It is evident that collectors of freshwater Algae in the Antarctic should in future pay particular attention to these huge strata of Phormidium and bring back material of them from as many diverse localities as possible.”

Further illustrating the taxonomical variety present within orange mats are the so‐called red mats (Figure 2c). Red mats are darker in color, have a more “slick” or “rubbery” appearance, and are often located in the stream between orange and black mats. Yet, whether or not the red subdivision is real or necessary remains an open question: Red mats are rare, occurring only in streams that reliably flow every summer (e.g. Canada, Popplewell, Lawson), and taxonomically differ in their dominant filamentous cyanobacteria, which generally have finer trichomes than those forming orange mats. Van Horn et al. (2016) suggested that the genus *Leptolyngbya* sets red mats apart, whereas Zoumplis et al. (2023) inferred that the differentiating genus is *Willmottia*. Given wide community differences already present among orange mats and given the similar habitat use among all “orange/red” mat types, it would be reasonable to group red mats within the larger “orange functional unit.” In contrast, these mats clearly look different and are composed of different biota and, thus, almost certainly contain ecological information because of that. We propose that a reasonable compromise is to collect and analyze them separately (as done here), with the rationale that you can always put data together, but you cannot always separate them once merged.

#### Black mats

Black mats are dark, dense growths that are loosely attached to the wetted sediments at stream margins, which correspond to the lateral extents of the hyporheic zone under “typical” flow conditions. As a result, hyporheic water is likely disproportionately important for keeping black mats hydrated as well as for supplying them with important solutes. Taxonomically, black mats are formed by the cyanobacterial genus *Nostoc* (Figure 2e–g). The name *Nostoc* itself is likely derived from a playful combination of old English and German words roughly meaning “nostril,” implying that these communities (accurately) look like blobs of snot (Potts, 1997).

*Nostoc* colonies are common occurrences worldwide (Dodds et al., 1995), often associated with episodically wetted or damp habitats, and are an important component to both Arctic and Antarctic freshwater ecosystems (e.g., Jungblut et al., 2021; Komárek & Elster, 2008). In the MDV, black mats have generally been reported as being formed by the species *Nostoc commune*, which is a putatively cosmopolitan species that exhibits a wide variety of morphological presentations depending on environmental characteristics (Novis & Smissen, 2006). Yet, studies on the diversity and phylogenetic position of Antarctic *Nostoc* have been limited (e.g., Wright et al., 2001), and although there is evidence of different genetic groups being present in the MDV (Novis & Smissen, 2006), it is not clear how unique or widespread these groups might be. Indeed, it is widely known that the genus *Nostoc* itself needs revision (Komárek et al., 2014), with many new species and new *Nostoc*‐like genera described in recent years (e.g. Bagchi et al., 2017; Pal et al., 2025). Therefore, the identity and distributions of *Nostoc* species composing MDV black mats should be investigated, especially in light of the emerging patterns of cyanobacterial endemicity in Antarctica (Durieu et al., 2024).

Regardless of which species make up black mats, what sets them apart functionally is their ability to fix atmospheric nitrogen. Although nitrogenase genes have been linked to several other groups of cyanobacteria (including from *Calothrix*, a common encrusting MDV GFS cyanobacterium) and whole mat types (Zoumplis et al., 2023), *Nostoc*‐based black mats likely fix more nitrogen from a mass‐balance perspective based on field rate measurements (Howard‐Williams et al., 1989). At the same time, more work is necessary to adequately constrain the magnitude and controls on these process rates given the usual “snapshot” approach to their measurement (e.g. Howard‐Williams et al., 1989; McKnight et al., 2007; Sohm et al., 2020). One notable complication is that black mats may exhibit temporal shifts in their nitrogen acquisition strategies, with nitrogen‐fixation rates more intense earlier in the melt season and urea breakdown rates greater later in the melt season (Zoumplis et al., 2023). Black mats also utilize dissolved inorganic nitrogen from streamwater, particularly where nitrogen concentrations are high, which is an energetically cheaper option than nitrogen fixation when feasible (Kohler et al., 2023) and, perhaps, makes nitrogen‐fixation rates spatially variable as well.

#### Green mats

Dense streamers of the Chrysophyte *Hydrurus foetidus* are a common site in GFSs worldwide (Hieber et al., 2001; Uehlinger et al., 2010; Ward, 1994), including in the mild and precipitation‐rich Maritime Antarctic (Elster & Komarek, 2003; Pizarro et al., 1996). Yet, in the MDV, this niche has seemed to be filled, rather, by chlorophytes, which constitute the basis of the communities referred to as “green mats” for sake of consistency. Green mats are the most heterogeneous of the three mat types in their distribution, given that they require a stable anchor to establish and grow in streams and that they create a tuft‐ or ribbon‐like macroscale morphology. Because of the need for larger‐sized substrata, their distribution is a function of the average grain size of sediment in the streambed, with greater green‐mat frequency (and biomass) in streams with greater average sediment sizes. When attached to the sides of rocks, green‐mat streamers can hold elevated positions above the sediments, potentially utilizing the water column more so than other mat types (although green tufts adhering to the undersides of rocks remain low in the water column). In these situations, with exposure to flowing water increasing nutrient delivery rates, the trailing filaments can visibly grow during the course of a summer season.

**FIGURE 3:**
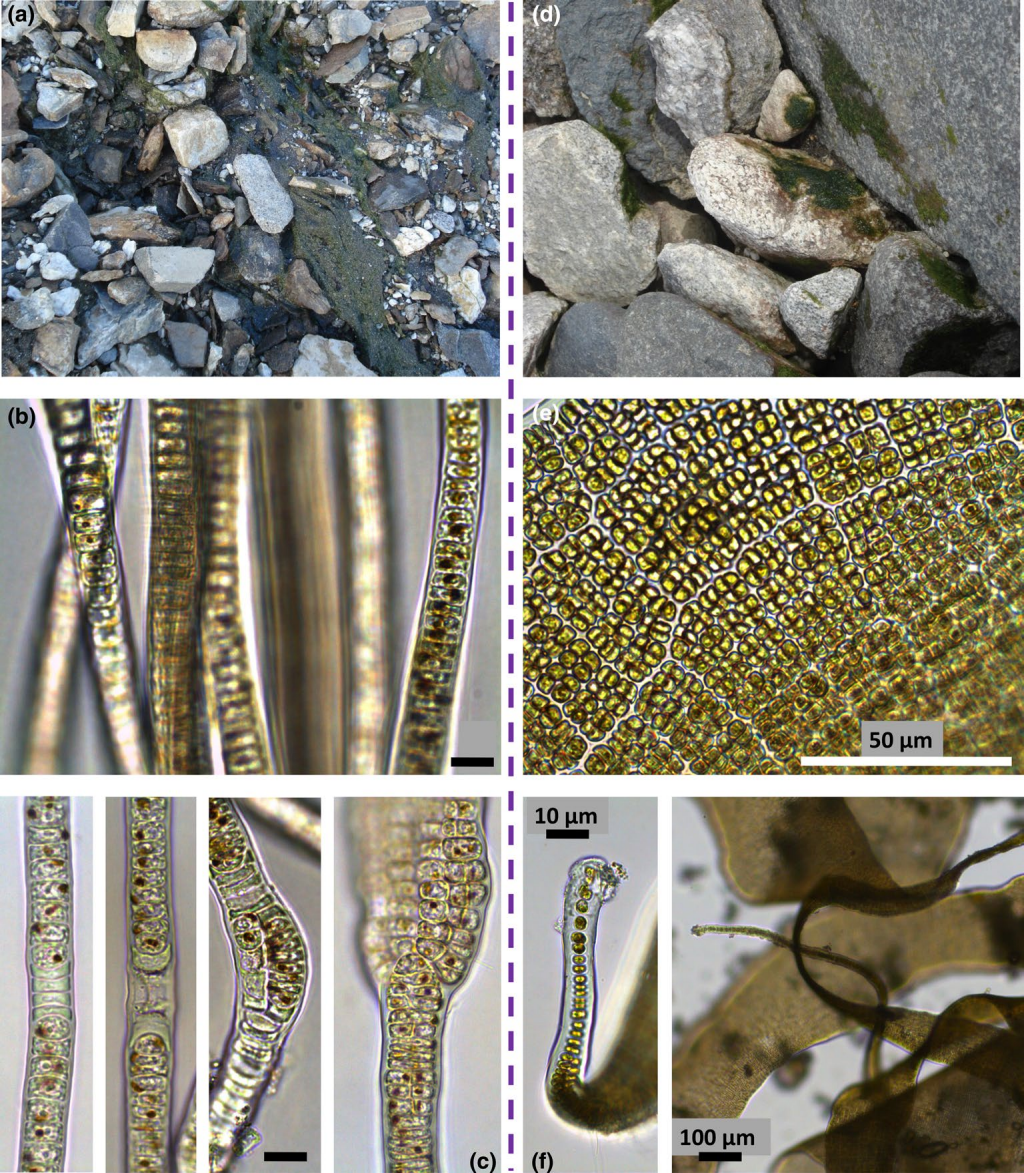
The green algal genus *Prasiola* often forms the basis of green mats, and here both “Hormidium” and “Schizogonium” stages are compared. In the left column, “Hormidium” stages are shown as they appear (a) in the field (from a tributary of Garwood Stream), (b) as well as under light microscope (from upper Von Guerard Stream), with (c) a series of images illustrating the separation of uniseriate filaments into multi‐seriate ones (from upper Von Guerard Stream). In the right column is pictured the “Schizogonium” stage (d) in the field (from Commonwealth Stream), (e) under light microscope (from Commonwealth Stream), and (f) at lower magnification depicting a holdfast (from Lawson Creek). The scale bar is 10 μm in (b) and (c) and as specified in (e) and (f). All images by T.J. Kohler.

Like orange mats, the foundation of green mats varies and can be coarsely divided into *Prasiola‐* and non‐*Prasiola*‐based types. *Prasiola* is a genus of green algae (phylum: Chlorophyta) from the class Trebouxiophyceae and is common in cold coastal areas worldwide (Figure 3). Perhaps because of this, and the fact that most stream research in the MDV has been focused on ecology rather than taxonomy, green growth in the MDV is often assigned to the genus *Prasiola* a priori. However, taxonomic identifications are also not straightforward for this group and are made even more difficult by *Prasiola*'s complicated life cycle. Take, for example, Fritsch (1917, p. 2), who wrote:

In view of the great abundance of *Prasiola crispa* in these collections, I was led to make a somewhat careful study of the filamentous states associated with it…these are sufficiently diverse, but I have not been able to convince myself that there are any actual specific differences between them – rather, they all seem connected by transition with one another.

Indeed, *Prasiola* has two main life stages: a “Hormidium” stage, which forms tough, asexually reproducing filaments through fragmentation (Figure 2a–c), and a “Schizogonium” stage, which produces small tufts on the underside of rocks and reproduces by aplanospores or oogamy (John et al., 2002, John, 2003; Figure 2d–f). The result is a physically divergent presentation at both micro‐ and macroscales (Richter et al., 2017). The Hormidium stage, especially, can get very dense, to the point that the term *mat* certainly becomes appropriate.

Within the genus *Prasiola*, *P. crispa* has been historically the most reported in the region (Fritsch, 1912, 1917; Seaburg et al., 1979; West & West, 1911). Given its cosmopolitan nature and its ubiquity and abundance in Antarctica in particular, *P. crispa* has served as a eukaryotic model organism for physiological experiments on the effects of ultraviolet (UV) radiation, salinity stress, and desiccation (Arzac et al., 2024; Jacob et al., 1991, 1992) and is an excellent indicator of biotic influence given its affinity for ornithogenic soils (Ohtani et al., 2000). In addition to *P. crispa*, both *P. antarctica* and *P. calophylla* have also been reported from the McMurdo Sound Region (e.g., Broady, 1982, 1989; Fritsch, 1912). Yet, definitive morphological identifications are difficult due to intraspecific variation. In one of the few papers investigating the molecular diversity of Antarctic *Prasiola*, Moniz et al. (2012) reported that while *P. antarctica* and *P. crispa* were indeed present in the McMurdo Sound Region (with caveats, as outlined in their paper), they also observed that the Antarctic specimens they had morphologically identified as *P. calophylla* were distinct from the *P. calophylla* inhabiting the Northern Hemisphere. They, then, described this taxon as a new species: *P*. *glacialis*. Although it is very difficult to differentiate these three *Prasiola* species morphologically, it is possible that *P. glacialis* is the most common species in MDV GFSs given its reported preference for intermittent oligotrophic streams rather than rookery soils, which is the preferred habitat of *P. crispa* and *P. antarctica* (Broady, 1989; Moniz et al., 2012).

Yet, *Prasiola* is not the only possible foundation for green mats. For example, Howard‐Williams et al. (1986) described “filamentous strands” of *Tribonema* sp. and *Binuclearia tectorum* in Adams Stream from Miers Valley, and Broady (1982) and Vincent and Howard‐Williams (1989) similarly reported “tufts and streamers” of *Binuclearia tectorum* from Canada Stream. (Both of these studies additionally reported *P. calophylla*.) In the few molecular investigations of MDV green mat diversity, Zoumplis et al. (2023), Van Horn et al. (2016), and Matula et al. (2025) all observed other green organisms serving as the basis for green mats. For example, Van Horn et al. (2016) observed that Taylor Valley green mats disproportionately consisted of coccoid *Chlorococcum* and *Chlorella*, whereas Matula et al. (2025) observed green mats from both Taylor and Miers Valley composed primarily of the genera *Hazenia* and *Pleurastrum*. Thus, it remains an open question how diverse the composition of MDV green mats can be. Nevertheless, their variable presentation and composition provide a good argument for the use of a color‐based functional grouping to investigate reach‐scale stream ecology rather than taxonomic‐based designations, depending on the research question.

### Elemental and isotopic composition

#### Elemental stoichiometry

**FIGURE 4:**
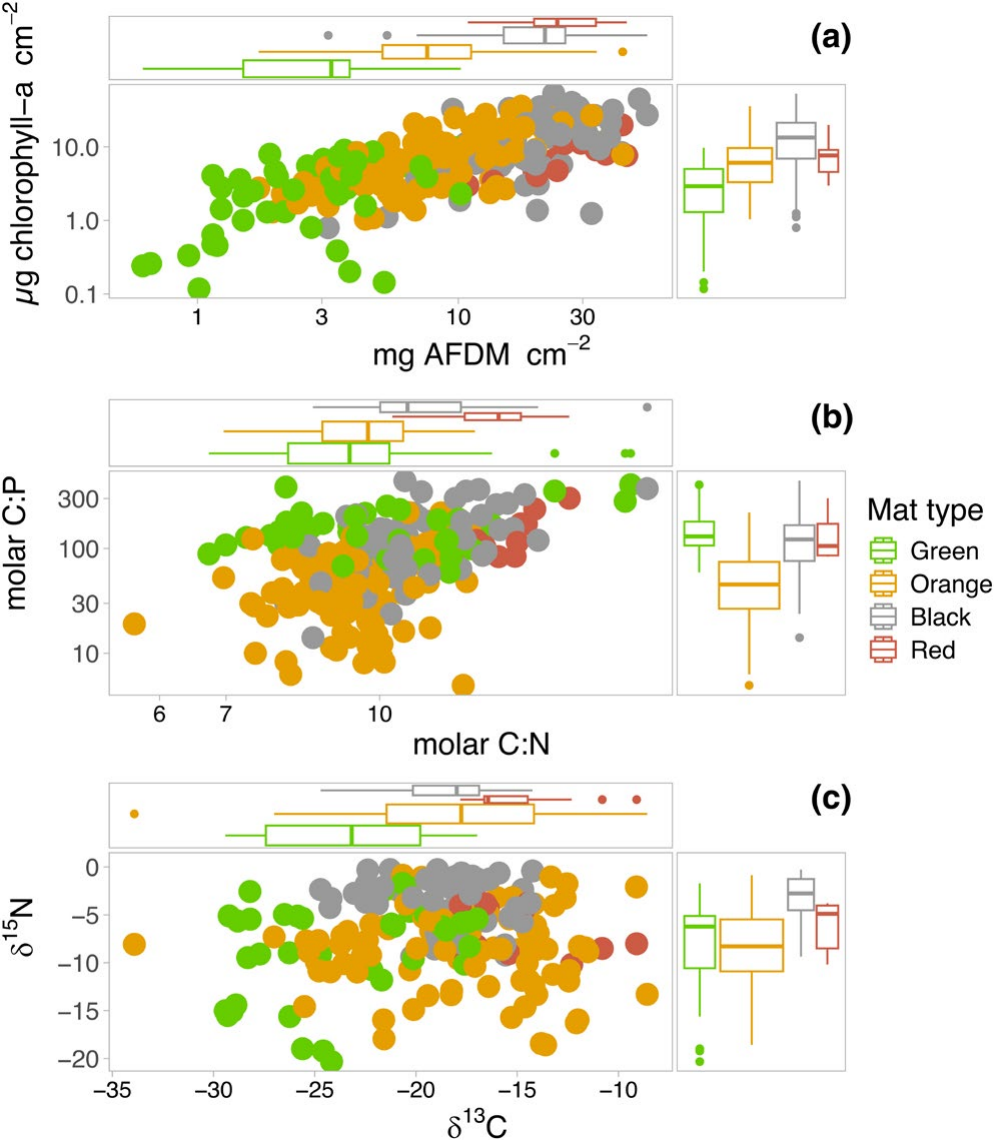
Stream mats show differences in their (a) biomass (measured as chlorophyll *a* and ash‐free dry mass [AFDM]), (b) molar C:N and C:P elemental ratios, and (c) *δ* ^13^C and *δ* ^15^N isotopic signatures, providing evidence of structural and chemical differences in addition to their contrasting appearance, taxonomic compositions, and in‐stream habitat. Data are plotted from Kohler and McKnight (2023).

Along with physical appearance, taxonomic composition, and stream subhabitat, mats can also be distinguished based on their biomass (as ash‐free dry mass, or AFDM, and chlorophyll *a*) and elemental ratios (molar carbon:nitrogen:phosphorus, or C:N:P). In terms of biomass, black mats consistently have the greatest AFDM and chlorophyll *a* values per unit area, followed by orange and, then, green mats (Figure 4a). In terms of elemental ratios, the three mat types are roughly comparable in their molar C:N (all with ratios between 9 and 11 across Taylor Valley; Kohler et al., 2023; Figure 4b), with values being slightly above the Redfield Ratio of ~7. In contrast, orange mats often have had lower C:P and N:P ratios than black and green mats (averaging ~50 for C:P and ~ 6 for N:P across Taylor Valley; Kohler et al., 2023) and are well below the Redfield Ratio for both (i.e., 106 and 16, respectively). Both black and green mat C:P ratios have averages between 130 and 140, and their N:P between 14 and 16—numbers that are very close to Redfield Ratio values. However, it remains an open question why orange mats have lower C:P and N:P ratios than other MDV mat types.

Algae in general can assimilate excess nutrients when available (i.e., “luxury consumption,” Sterner & Elser, 2002). However, cyanobacteria, specifically, can take up excess P and store it for later use within intracellular polyphosphate bodies (Dodds et al., 1995; Jentzsch et al., 2023; Stal, 2000), whereas N can be stored using protein‐based phycobilisomes, which can represent up to half of the cell's N, and be disassembled during periods of N limitation (Grossman et al., 1993; Krauspe et al., 2021). Thus, ambient nutrient concentrations (and perhaps even occasional nutrient pulses) can influence nutrient storage and, ultimately, mat elemental ratios. In their investigation into how nutrient availability compared with mat C:N:P ratios, Kohler et al. (2023) showed that higher streamwater N and P concentrations were associated with lower C:N and C:P ratios for orange mats across Taylor Valley. No significant relationships, however, were observed for black or green mats. Although it is not entirely clear why that was the case, it is possible that the close proximity of orange mats to hyporheic upwelling zones may allow them better access to recently weathered P and mineralized N compared to other mat types. Although black mats should also have access to hyporheic water given their position at stream margins, slower delivery rates and a more limited surface area exposed to streamflow may comparatively limit their uptake and storage. Interestingly, Kohler et al. (2023) observed that Taylor Valley black mat C:N ratios were lowest near the Ross Sea coast, where dissolved inorganic N concentrations are low and the coverage of black mats high, suggesting that black mats are better able to utilize the N fixed in their surroundings than N from the stream.

The elemental composition of mats may further reflect variability in flow conditions. For example, stream mats elsewhere in Antarctica have been shown to exhibit lower C:P and N:P ratios than mats from non‐flowing habitats such as soils and ponds (Fernandez‐Valiente et al., 2007). This is likely because constant flow (such as that experienced by orange and green mats) removes senescent cells and reduces boundary layer size, thereby increasing nutrient delivery to cells and promoting lower ratios (Davey, 1993; Rochera et al., 2013). By comparison, when black mats are inundated by streamwater (and assuming they are not exported), they may have greater boundary layers due to greater biomass, which could comparatively hinder nutrient uptake. These same mechanisms may also operate over intra‐ and interannual timescales. For example, Kohler, Chatfield, et al. (2015) observed that in one stream (i.e., Green Creek), orange mat C:N and C:P ratios were lower following a summer with higher and more consistent flow than one where flow was overall lower and more intermittent. Although it is unclear if these patterns would hold over greater spatial and temporal scales or among the different mat types, these results support the notion that greater nutrient delivery and uptake comes with more, and more dependable, discharge.

#### Stable isotopes

Mats are also distinct in their stable isotopic (δ^13^C and δ^15^N) signatures (Figure 4c). Most prominent are the black mats, which are consistently enriched in δ^15^N and near the atmospheric standard (~0‰) and indicative of N_2_‐fixation (Kohler et al., 2018, 2023). Meanwhile, orange/red and green mats range from 0 to <−20‰ across Taylor Valley (Kohler et al., 2023). Given the depleted nature of MDV atmosphere‐derived N (Wada et al., 1981), these δ^15^N values reflect the two main sources of N: atmospheric deposition onto glaciers and black mats. For example, when black mat biomass is high and glacier‐derived N low, more stream N may originate from black mats, resulting in all mat types having δ^15^N signatures of ~0‰. Conversely, where black mats are scarce and dissolved N concentrations high, δ^15^N signatures of all mat types (including black mats) are more depleted (Kohler et al., 2023). Although mat δ^15^N enrichment could arguably also arise from fractionation associated with repeated downstream cycling (Michener & Lajtha, 2007), this would likely have a small impact on mat signatures overall given the large divergence of these endmember values and the fact that mat δ^15^N values are never substantially greater than ~0‰, which would be expected if intense cycling was the cause of enrichment. Because of these distinct sources of N and their correspondingly distinct signatures, MDV GFSs are particularly good ecosystems in which to study N cycling in streams.

In terms of δ^13^C signatures, green mats are consistently more depleted (averaging −24‰ across Taylor Valley) than black and orange mats (both having averages near −17‰), although the variability for orange mats specifically has been large (from −5 to −30‰; Kohler et al., 2023; Figure 4c). These differences may be rooted in both the taxa that comprise the mats and in habitat use. For example, the exopolymeric substances composing cyanobacterial sheaths are typically enriched in δ^13^C compared to bulk cell material (van Dongen et al., 2002), and C‐concentrating mechanisms may lead to further enrichment (e.g., Badger et al., 2002; Hanson et al., 2014), potentially explaining why the δ^13^C signatures of orange and black mats are more enriched (e.g., Rochera et al., 2013; Vuorio et al., 2006). Boundary‐layer size may also affect δ^13^C signatures by controlling CO_2_‐diffusion rates (Des Marais & Canfield, 1994; Keeley & Sandquist, 1992). Thus, by reducing boundary layers and stimulating atmospheric exchange, high flow velocities may encourage δ^13^C depletion (Finlay et al., 1999; Finlay & Kendall, 2007), whereas high biomass and sluggish flows may enrich signatures (Hill et al., 2008; Laws et al., 1995; Schouten et al., 2001). Therefore, depleted green mat δ^13^C signatures may partially be a function of their lower biomass, streamer‐like morphology, and water column utilization (e.g., Rochera et al., 2013). Lastly, δ^13^C signatures may also result from alternation between HCO_3_^−^ and CO_2_ as an inorganic C source. Mats may be better able to isotopically discriminate CO_2_ than HCO_3_^−^ (Des Marais & Canfield, 1994), and thus, the isotopic signature of HCO_3_^−^ may have a large influence on mat δ^13^C signatures when CO_2_ is limiting (Des Marais & Canfield, 1994; Finlay, 2004). Interestingly, the MDVs have had some of the highest δ^13^C values for HCO_3_^−^ ever reported for streams (especially up‐valley, Lyons et al., 2013), and it may therefore be unsurprising that these high values are also reflected in the δ^13^C signatures of some orange mats.

### Controls on mat presence, biomass, and coverage

#### Geomorphology

Given the distinctive nature of MDV GFSs and their inhabitants, it has remained a long‐standing question as to what factors control the presence, biomass, and coverage of different mat types. Most commonly, stream geomorphology has been given as the first‐order constraint on mat‐type distribution (Fountain et al., 1999). As discussed above, the three mat types are predictably observed in different zones or subhabitats of MDV streams: Orange mats cover the mainstem sediments, black mats are situated over hydrated stream margins, and green mats have attached to cobbles. As a consequence of landscape characteristics alone, not all mat types can inhabit all streams. For example, if streambanks are too incised and steep, such that adjacent sediments are actively eroding or cannot be wetted, black mats cannot form. Accordingly, black mat biomass was previously shown to be limited by the available “marginal wetted area” (Howard‐Williams et al., 1989). Similarly, if substrata are too fine, green mats have nothing to attach to, and unstable streambeds with rapid flow and high sediment loads have hosted sparse orange mats (Hawes & Brazier, 1991; Vincent & Howard‐Williams, 1986). In such a way, geomorphology dictates habitat availability and, in turn, mat‐type diversity. In Taylor Valley, more mat types have been observed in a given stream on average near the coast, where gradients are gentle, sediment sizes intermediate, and streambeds stable. Mat‐type diversity decreases upvalley toward Taylor Glacier, where streams are shorter, gradients steeper, and sediments either very large or fine and unconsolidated. These patterns have been temporally stable, with the same mat types observed in the same reaches year after year (Crisp, 2015; Kohler, Stanish, et al., 2015).

**FIGURE 5:**
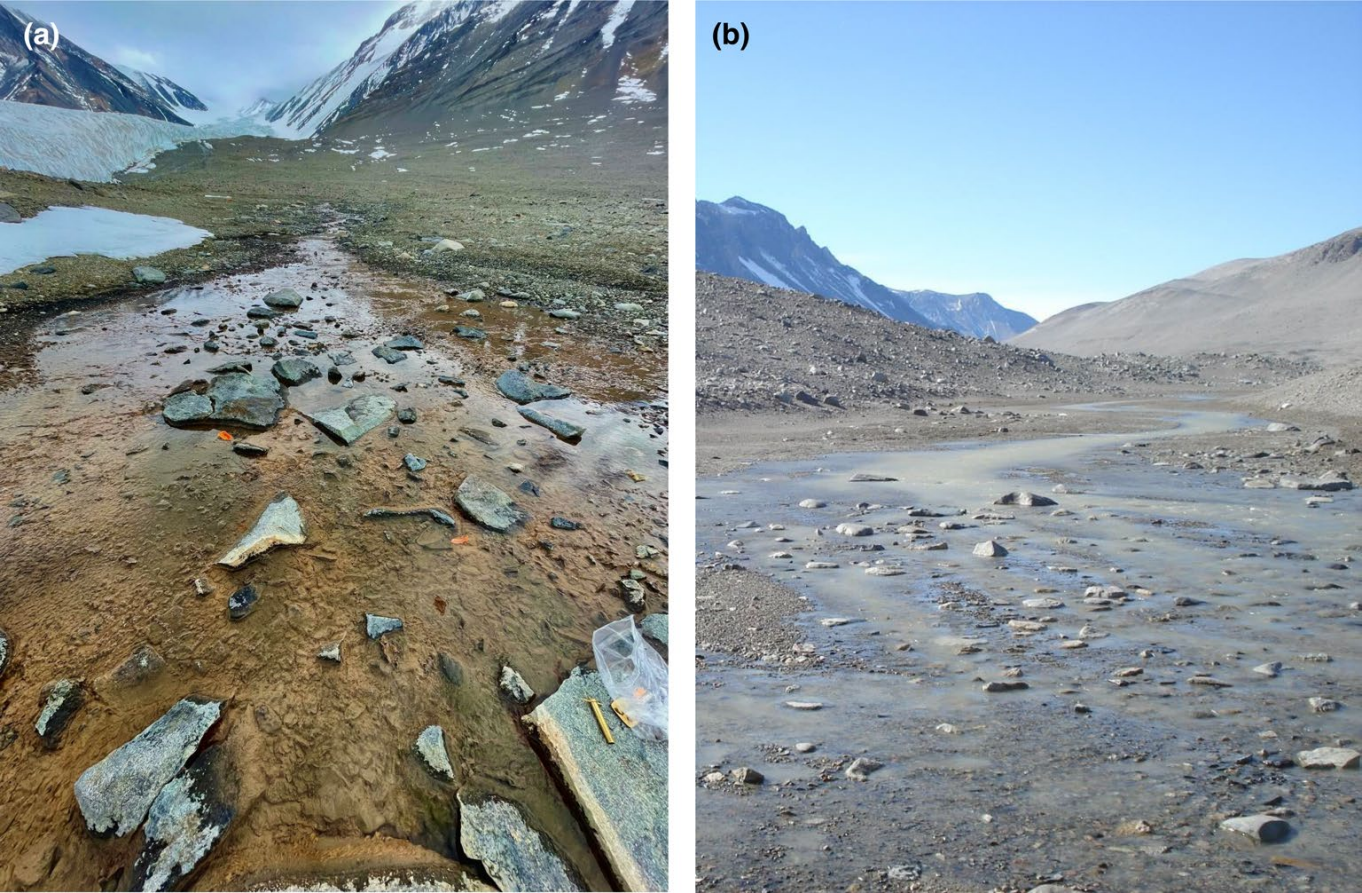
Within streams, orange mats occupy the main channel, black mats are observed at the margins, and green mats are attached to cobbles. The abundance of mats in streams is determined by a combination of geomorphology, suspended sediment loads, and hydrology. “High coverage” streams (a) have abundant mats thanks to their stable substrata, low suspended solid concentrations, and regular flows, whereas “low coverage streams” (b) have few mats, usually due to unstable streambeds and high sediment loads. Left image is a photo of Canada Stream (courtesy A. Wright), and right image is a photo of Priscu Stream (T.J. Kohler).

Geomorphology is also an important consideration for mat biomass and coverage. For example, some MDV streams (with appropriate slopes, sediment sizes, and sufficient freeze/thaw cycles) develop stable stone pavement streambeds (McKnight et al., 1999). The resulting stability, along with high stream width‐to‐depth ratios, promotes the accumulation of mat biomass (Howard‐Williams et al., 1986, 1989; McKnight et al., 1998). As a result, some streams (e.g., Canada, Green, and Bowles) have such extensive mat biomass that it is difficult to find an unoccupied patch of streambed. Such streams are referred to as high coverage streams (Figure 5a). Conversely, many MDV streambeds have been composed of fine unconsolidated sediments (especially those draining alluvial areas, e.g., Lost Seal and McKnight), resulting in unstable substrata and high suspended‐sediment concentrations. Elevated suspended sediment loads are particularly problematic because when coupled with sufficient discharge, they produce a scouring “belt‐sander” effect, which leads to the sloughing of existing biomass and prevents the colonization of new growth (Vincent & Howard‐Williams, 1986). In such streams, mats have been patchy in distribution and generally restricted to side channels and backwaters, if they have been present at all (Howard‐Williams et al., 1986). Accordingly, these streams are referred to as “low coverage” streams (Figure 5b). Like for mat‐type diversity, almost all high‐coverage transects in Taylor Valley are located within the Fryxell Basin near the coast, while upvalley toward the Bonney Basin, streams are mostly low coverage (Alger, 1997; Kohler, Stanish, et al., 2015). These high/low coverage designations are useful for understanding long‐term patterns in stream‐mat abundance, since streams of different geomorphologies (and thus coverages) may not respond uniformly to hydrological change.

#### Hydrology

Flow is widely considered to be the master variable in stream ecology. In the MDV, the absence of meaningful groundwater (thanks to continuous permafrost) and precipitation contributions means that stream discharge almost exclusively originates from glacial meltwater (Wright, Gooseff, et al., 2024). Therefore, like in other GFSs globally, MDV stream discharge is inextricably related to solar radiation, glacier size, and glacier aspect. In general, MDV streams flow for about 8–10 weeks from November to February, although the exact timing and duration differs by individual stream as well as yearly with weather. Stream length also influences the timing, duration, and extent of flow. For example, the relatively short Canada Stream flows into Lake Fryxell regularly all summer, whereas water carried by the much longer Von Guerard Stream may reach the same lake for the first time well into the melt season and may only flow for a short time thereafter (Fountain et al., 1999; McKnight et al., 1999; Wlostowski et al., 2016). As a result, hydrological variability manifests both spatially (i.e., across streams) and temporally (both intra‐ and interseasonally), representing a core challenge for MDV biota.

Discharge can vary widely over the course of a day, with daily flow peaks coinciding with the greatest intensity of solar radiation on glaciers (Conovitz et al., 1998). Given differences in glacier aspect, slope, and stream length, each stream's peak flow occurs at a different time of the day (Wlostowski et al., 2016), and discharge can increase by more than an order of magnitude during these times. The resulting flow pulses can be highly disruptive, efficiently mobilizing and exporting biomass, and represent a major loss mechanism for stream mats. As modeled by Cullis et al. (2014) for Von Guerard Stream, concentrations of mat biomass (as particulate organic matter) were greatest on the rising limb of each flow pulse, resulting in a clockwise hysteresis pattern indicative of supply limitation. This result, even in a stream with abundant mat biomass, was consistent with the generally cohesive nature of the mats. Given that the bulk of organic matter is mobilized and exported during these punctuated periods each day, flow pulses are particularly important for understanding local biogeochemical cycles and organism dispersal (Stanish et al., 2024). Furthermore, since mats represent multiple seasons of growth (Kohler, Chatfield, et al., 2015; Vincent & Howard‐Williams, 1986), flow events of exceptional magnitude can potentially influence how much mat biomass is present in future years.

At the other extreme, streamflow can also turn off suddenly with cloudy weather, meaning that mats can also face prolonged and repeated desiccation within the flow season. Such interruptions represent missed opportunities for growth, and indeed, Kohler, Chatfield, et al. (2015) observed that in Green Creek, a summer with more hydrological interruptions resulted in less mat growth than one with more consistent flow. Furthermore, it is also possible that in some low‐flow summers, mats inhabiting the lower reaches of longer streams (e.g., Von Guerard, Delta) never get the opportunity to reactivate. Like for high‐flow periods, low and interrupted streamflow may have impacts on mat biomass that transcend summers. In support of this, successive low flow summers in the MDV were observed to correspond with decreases in mat biomass, whereas years with more stable and elevated flows led to increases in mat biomass over a span of 2 decades (Gooseff et al., 2017; Kohler, Stanish, et al., 2015). Such results indicate that mat biomass from any given year is directly a function of surviving mat biomass from previous years.

#### Cold and desiccation

Cold and dry conditions make up the bulk of an MDV year. As a result, physiological adaptations for both desiccation and low temperatures are essential for resident organisms (McKnight et al., 1999). Mats persist in freeze‐dried dormant states during the winter, appearing as crusts within dry streambeds (e.g. Howard‐Williams et al., 1989; Vincent & Howard‐Williams, 1986; Vincent, Castenholz, et al., 1993; Vincent, Downes, et al., 1993; Figure 2a,e). These crusts in turn provide the basis for the next year's growth, ensuring "pre‐emptive competitive success" when flow returns and, ultimately, allowing for the perennial nature of mats (Vincent, 2007). Amazingly, freeze‐dried cells can remain viable for years, perhaps millennia (Antibus et al., 2012; Zaikova et al., 2019), and cells resume metabolism almost immediately following rehydration (e.g. Vincent & Howard‐Williams, 1986). In perhaps the largest‐scale rehydration experiment in MDV history, McKnight et al. (2007) experimentally diverted flow from upper Von Guerard Stream into an abandoned (“Relict”) channel, which had been dry for about 2 decades. Within a week of rehydration, mats were present at high biomasses, exhibiting elevated rates of photosynthesis and N fixation. However, the efficiency of this ability is likely mat‐type specific, with black mats (which are routinely outside streamflow and thus potentially undergo more freeze/thaw cycles per summer) fully recovering original photosynthetic and respiration rates within ~10 min, whereas orange mats may take days to do the same (Hawes et al., 1992).

One of the keys to mats tolerating dry and freezing conditions lies in their production of extracellular polymeric substances, which in the case of *Nostoc*, specifically, represent a large part of the colony by volume (Potts, 2000). Extracellular polymeric substances are hydrophilic and provide a protective layer around cells that can retain moisture until flow resumes or, otherwise, allow cells more time to prepare for what could be prolonged dry conditions (Potts, 1994, 1999). Extracellular polymeric substances also protect cells against membrane fusion and leakage, both of which are major issues associated with freezing and desiccation (Hill et al., 1997). Furthermore, the production of cryoprotectants such as trehalose is a widely employed adaptation to prevent osmotic stress, and the genes responsible have been previously identified in sequences from black (Jungblut et al., 2021) and orange (Varin et al., 2012) polar mats. Lastly, here and in similar systems, cold‐shock and antifreeze proteins promote the long‐term persistence of mats by helping them to adapt to temperature changes and preventing crystal formation (Busi et al., 2022; Varin et al., 2012; Zoumplis et al., 2023). Interestingly, in antifreeze activity assays (determining the distance between melting and freezing temperature), the greatest levels of thermal hysteresis were also observed in black mats, followed by green mats and, then, red/orange mats (Zoumplis et al., 2023).

#### Radiation

Due to a general lack of protective snow cover, MDV mats are exposed to high levels of UV radiation when the sun reemerges in spring. Given that the onset of streamflow may still be many weeks away, this exposure to potentially damaging irradiance while still in a physiologically inactive state creates a particularly challenging photobiological environment. Furthermore, when streamflow finally returns, the high water clarity offers little protection. In contrast to most GFSs globally, many MDV streams are clear‐flowing, lacking the high concentrations of subglacially derived weathering products that very efficiently attenuate and limit light elsewhere (Fountain et al., 1999; Kohler et al., 2022). As a result, mat growth can be inhibited by UV radiation, although individual mat taxa respond differently to this stress (Vincent, Castenholz, et al., 1993; Vincent & Quesada, 1994). For example, while UVB radiation was observed to inhibit the growth rate of the Antarctic cyanobacterium *Phormidium murrayi* (now revised as *Wilmottia murrayi*; Strunecký et al., 2011) by 62%, it completely shut down growth for *Oscillatoria priestleyi* (now revised as *Phormidium pseudopriestleyi*; Anagnostidis & Komárek, 1988; Vincent & Quesada, 1994). Given the potential importance for mat growth and structure, Vincent and Quesada (1994) outlined four main mechanisms that mat organisms employ to cope with UV radiation: (1) screening, (2) avoidance, (3) quenching, and (4) repair.

In terms of screening (1), mats inhabiting exposed areas require an overstory of protective pigments (e.g., scytonemin and mycosporine‐like amino acids) that absorb in the UV wavelengths (Vincent, Downes, et al., 1993). *Nostoc*, for example, is exposed to high levels of solar radiation given its position at the edge of streams. To cope with this, it places the dark pigment scytonemin within the upper parts of its mucilaginous envelope, which gives colonies their characteristically dark coloration (Dodds et al., 1995; Vincent, 2007). In terms of avoidance (2), studies have shown that some Oscillatorian cyanobacteria migrate upward with lower UV intensities and downward under higher intensities (Nadeau et al., 1999; Vincent, Castenholz, et al., 1993). Interestingly, as *O. priestleyi* (noted above) apparently does not contain common protective pigments (Vincent & Quesada, 1994), it perhaps compensates for this through enhanced motility. Similarly, green mats (which lack an overstory of protective pigments) take cover beneath boulders (Broady, 1989), presumably to prevent DNA damage. Quenching (3) refers to strategies for dissipating harmful photochemical energy, generally via specialized enzymes and pigments (e.g., Roos & Vincent, 1998; Vincent, Downes, et al., 1993; Vincent & Quesada, 1994). Carotenoids (e.g., β‐carotene) in particular can act as antioxidants against free radicals, and both carotenes and xanthophylls have their highest concentrations at mat surface layers (Vincent & Quesada, 1994), resulting in vivid yellow–orange–red colorations and substantially reducing incident irradiance at the depth where most photosynthesis in the mat occurs (Hawes et al., 1999; Vincent, Downes, et al., 1993). The last mechanism is repair (4), which consists of several sub‐mechanisms: photoreactivation, nucleotide excision repair/dark repair, Photosystem 2 repair (D2 protein resynthesis) after bright light damage, and recombinational repair (Vincent & Quesada, 1994). Although not the focus of any MDV stream studies to our knowledge, studies on both MDV soil microbes (Rzoska‐Smith et al., 2023) and GFSs biofilms worldwide (Busi et al., 2022; Michoud et al., 2025) have observed genes related to oxidative stress management and DNA repair. Importantly, repair (as well as quenching) requires an active physiology, which in turn requires water, and is made less efficient by low temperatures (Vincent, 2007). Thus, the prevalence of avoidance mechanisms in particular is consistent with the need to survive high irradiance while cold and dry.

#### Elevated temperatures

Very much related to irradiation is temperature. In the MDV, streamwater temperatures are generally only slightly above 0°C at glacier termini, but may reach 10–15°C near the outlets of longer streams on a clear sunny day, with daily differences of 6–9°C recorded (Cozzetto et al., 2006, 2013). Diel changes in stream temperature also drive changes in the extent of hyporheic exchange processes that bring nutrients and other solutes into the stream. As a result, communities living within the same streams may be exposed to different temperatures and other water‐quality conditions depending on the month, time of day, and longitudinal location. Given changes in diffusion rates and viscosity that come with temperature changes, this is likely to be a highly relevant consideration for stream organisms and their metabolisms.

Previously, the optimum temperature for *Nostoc* C fixation (in MDV soils), as well as for cultures of Antarctic cyanobacteria and *Prasiola*, was observed to be nearly 20°C for all (Arzac et al., 2024; Novis et al., 2007; Roos & Vincent, 1998; Tang et al., 1997), much higher than what would be expected given their habitats of origin (Seaburg et al., 1981). Accordingly, orange and green mats are not likely to reach their optimal temperatures often (if ever) given their submergence in streams, and thus, near‐freezing temperatures likely represent a growth and metabolic constraint for these communities (Vincent & Howard‐Williams, 1986). At the same time, MDV air temperatures can reach 10°C in summer, and soil surface temperatures can reach >25°C (Obryk et al., 2020). Thus, black mats may absorb considerable radiation given their location outside the streambed and their dark coloration.

Importantly, Vincent and Howard‐Williams (1989) observed that rates of photosynthesis, respiration, and biosynthesis all increased for Canada Stream mats over a representative temperature gradient (0–10°C). They also observed, however, that higher temperatures disproportionately increased respiration over photosynthesis, which was suggested to represent increases in the decomposition rates of senescent mat material. At the same time, several authors have also argued that these high biomass stream mats are mature canopies wherein autotrophic and heterotrophic biomass reach a level of equilibrium, and the community becomes balanced with respect to C exchange (e.g. Hawes, 1993). Thus, while increasing air and water temperatures will probably not unilaterally promote organic matter production in the MDV, particularly in high biomass mat communities, it remains unclear if a changing climate will promote overall C accumulation or loss in the region.

#### Carbon and nutrients

While anthropogenic nutrient inputs are mostly lacking in the MDV, some streams can exhibit surprisingly high concentrations depending on their location, the time of year, and the available nutrient sources. Here, important elements such as P and silicon (Si) are made available through the weathering of apatite and silicate minerals in the streambed, respectively, and enter surface water through hyporheic exchange (Gooseff et al., 2002; Heindel et al., 2018; Maurice et al., 2002). Inorganic N, in contrast, is added primarily via atmospheric deposition onto glaciers, entering streams via glacial melt. Ionic concentrations in streamwater are greatest during first flows, when solutes accumulated over winter are flushed (“austral spring freshets”; Downes et al., 1986; Vincent & Howard‐Williams, 1986; McKnight et al., 2007). Thereafter, weathering‐derived ions (as well as others) have generally exhibited chemostatic concentration–discharge relationships (Singley et al., 2021; Torrens et al., 2022; Wlostowski et al., 2018).

The organic forms of C and nutrients are highly variable within and across sites, and five main sources have previously been identified (Downes et al., 1986; Moorhead et al., 1999): (1) autochthonous organic matter fixed in the streambed, which further generates coarse and fine particulate organic matter (CPOM and FPOM; Cullis et al., 2014; Stanish et al., 2024); (2) wildlife (of negligible importance in the MDV, aside from the occasional seal or penguin mummy); (3) erosion of legacy materials, such as ancient lacustrine and marine sediments; (4) streambed sediments, where organic matter from previous seasons may be stored; and (5) the source glaciers, accounting for atmospheric deposition and accumulation onto both ice and snow. These neat categories of sources, coupled with limited hydrological flowpaths, make MDV streams excellent model systems to study lotic biogeochemical cycles.

Given that the upvalley portion of Taylor Valley is older, it has arguably had more time to accumulate nitrate (Barrett et al., 2007). This age, coupled with relatively short streams that limit microbial uptake, can help to explain the greater streamwater N concentrations upvalley (Kohler et al., 2023; Welch et al., 2010). In contrast, as P is continuously weathered from apatite in the hyporheic zone, the longer streams overlying the younger soils of the Fryxell Basin promote higher dissolved P concentrations downvalley (Barrett et al., 2007). As a result, streamwater N:P ratios are lower near the coast and higher toward Taylor Glacier (Kohler et al., 2023; Welch et al., 2010). Kohler et al. (2016) confirmed that these N:P ratios indeed have biological implications, showing that greater mat growth accumulated on N‐amended artificial substrates in two Fryxell Basin streams rather than on P‐amended substrates or controls. Although unlikely to be the primary reason, N limitation could potentially have an influence on the distribution of mat types as well: N‐fixing black mats have their greatest biomass and coverage in the N‐limited Fryxell Basin, where they would theoretically have a competitive advantage.

Streamwater nutrients are inextricably related to stream mats in that nutrient availability (partially) dictates mat biomass, and mat biomass, in turn, influences concentrations. In the Fryxell Basin, past work has shown that glacier‐derived N can be removed quite rapidly (Gooseff et al., 2004; Koch et al., 2010), and N concentrations have remained relatively low thereafter (e.g. Howard‐Williams et al., 1989). Indeed, high‐coverage stream reaches generally have had lower nutrient concentrations than low‐coverage reaches (McKnight et al., 2004). Yet, mat biomass can remain high even after many kilometers, resulting in a mat‐nutrient paradox: How is it possible to support downstream biomass when most N was already removed upstream?

Although the continual downstream weathering of apatite may help to counterbalance microbial uptake of P, a combination of N_2_ fixation and mineralization of upstream mats is needed to facilitate mat growth downstream (Howard‐Williams et al., 1989; Kohler et al., 2018, 2023). By studying the δ^15^N isotopic composition of suspended particulate organic matter (Kohler et al., 2018), as well as the diatom assemblages observed therein (Stanish et al., 2024), it has been demonstrated that black mats are routinely inundated during daily flow peaks, mobilizing into streamflow, and transported downstream. Once mobilized, black mat material is either deposited on the streambed where it is processed by microbes directly (Zoumplis et al., 2023) or subducted into the hyporheic zone for remineralization (Heindel et al., 2021; Singley et al., 2021). The N liberated from mats is stored in the hyporheic zone (Singley et al., 2021, 2023), where dissolved N concentrations are noticeably higher than in the overlying water column (McKnight et al., 2004), and is released at upwelling zones downstream. Thus, black mat N fixation, mobilization, and mineralization is an important mechanism for balancing MDV stream N budgets.

#### Heterotrophic activity and species interactions

Although microorganisms make up the majority of biomass in all aquatic systems, this dominance is even more pronounced in MDV streams. Notably, all of the classic stream vertebrate and macroinvertebrate groups are absent here (i.e., fish, amphibians, insects, mollusks, crustaceans, etc.). Although other facultative aquatic invertebrates have been recorded (e.g., the springtail *Gomphiocephalus hodgsoni* and mite *Stereotydeus mollis*) and have been known to ingest cyanobacteria and diatoms (Davidson & Broady, 1996), their distributions have been patchy and biomass low (e.g., Nolan et al., 2006). Furthermore, although the MDVs are infamous for their rotifers, tardigrades (*Acutuncus antarcticus*), and nematodes (e.g., *Scottnema lindsayae*, *Plectus murrayi*, *Geomonhystera antarcticola*, *Eudorylaimus antarcticus*), which collectively represent the top of the local food chains (Shaw et al., 2018), their potential ecological role in streams has been minimally investigated.

In one of the few studies on the topic, Treonis et al. (1999) observed invertebrate abundances overall were greater in streams than dry soils. Therein, two nematodes (*Plectus* and *Eudorylaimus*), tardigrades, and rotifers were the most abundant, whereas the nematode *Scottnema* was absent in the stream sediments, seemingly preferring dry soils. Although these organisms presumably feed on mats (Shaw et al., 2018), grazing has historically been considered negligible as a loss mechanism due to very low grazer to mat biomass ratios. Yet, it is also true that this assumption lacks extensive testing, and thus, it remains an open question if grazers are capable of exerting top‐down control on mats generally or, at least, for certain mat types (Andriuzzi et al., 2018; Aranda, 2024). For example, there is some indication that grazers may show preference for some mat types over others, with *Prasiola* suggested to be too tough (or otherwise unpalatable) for springtails (Davidson & Broady, 1996). Furthermore, in Canada Stream, rotifers were observed to be most commonly associated with orange/red mats, nematodes with black mats, and tardigrades with green mats (Zoumplis et al., 2023).

Non‐Cyanobacterial bacteria (particularly within the Proteobacteria and Bacteroidetes) also constitute a non‐trivial proportion of mat diversity (Zoumplis et al., 2023). Yet, they have been scarcely investigated in MDV streams despite their putatively critical functional roles. Chief among these are heterotrophic pathways, which remove organic C (and thus mat material) from the system through decomposition, as well as return organic nutrients to inorganic forms. Glacier‐fed stream bacteria and archaea may also have very different and diverse chemotrophic metabolisms, utilizing a variety of molecules depending on the redox conditions present, and may even vary as a function of vertical gradients present in stream sediments or the mats themselves. Such metabolisms could lead to the oxidation and reduction of ions composed of N, sulfur, and iron, among others (Michoud et al., 2025). Furthermore, and despite decades of supposition otherwise, non‐Cyanobacterial bacteria may also be responsible for non‐trivial proportions of phototrophy, as has been inferred in Arctic mats (Vigneron et al., 2018). Through these functions, bacteria can almost certainly influence mat community structure through species interactions (e.g., competition, facilitation), even across domains (Stanish, O'Neill, et al., 2013), although the magnitude of these effects and the mechanisms responsible await elucidation.

Fungi are another group critical to stream ecosystem functioning that has received limited work in MDV streams. Ascomycota and Basidiomycota were previously observed in all mat types, though the latter was reported to have greater relative abundances among orange mats, and the former more abundance overall (Van Horn et al., 2016). Elsewhere in Antarctica, Basidiomycota have been implicated in forming discolored rings, or “blights,” upon mats, although the cause, geographical distribution, and temporal frequency of such events remain unknown (Velázquez et al., 2016). Meanwhile, Chytridiomycota (or “chytrids”) were also reported (Van Horn et al., 2016). This group has recently garnered attention for acting as algal parasites, potentially stimulating organic‐matter cycling in GFSs in which algae are the dominant source (Kohler et al., 2022). Lastly, phages represent critical components to cryospheric communities, with a previously demonstrated importance to glacier surfaces (Perini et al., 2024), Arctic *Nostoc* mats (Jungblut et al., 2021), alpine GFS biofilms (Peter et al., 2024), and MDV outlet lake communities (Lisle & Priscu, 2004; Säwström et al., 2008), the latter of these exhibiting host‐ and basin‐specific distributions (Michoud et al., 2025; Robinson et al., 2024). It is therefore unlikely that viruses would be of any less importance to MDV GFS mats. Collectively, non‐Cyanobacterial bacteria, archaea, fungi, and phages represent understudied though vitally important components of MDV biological diversity and biogeochemical pathways.

## STREAM DIATOMS

### Model organisms for community assembly

**FIGURE 6:**
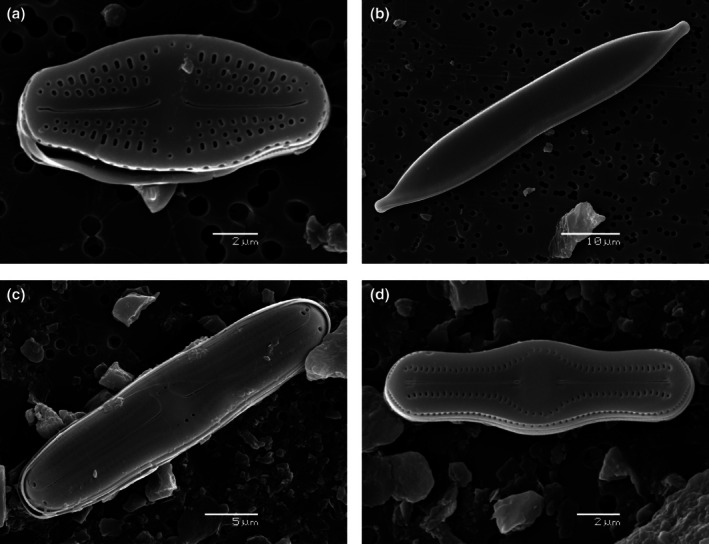
McMurdo Dry Valley stream diatom communities are dominated by aerophilic taxa. Pictured here are scanning electron microscope images of four characteristic genera: (a) *Luticola* (*L. austroatlantica*), (b) *Hantzschia* (*H. amphioxys*), (c) *Muelleria* (*M. meridionalis*), and (d) *Humidophila* (*H. australis*). Micrographs from (a) and (b) are from Bohner Stream, while (c) and (d) are from the Onyx River near Lake Vanda.

Diatoms (Bacillariophyceae) are a common component of GFS mats globally (e.g., Rott et al., 2006; Uehlinger et al., 2010) and represent a promising model group to study protist evolution, ecology, paleolimnology, and biogeography (Pinseel et al., 2024), especially within Antarctica (Spaulding et al., 2010; Verleyen et al., 2021). At present, ~50 morphotypes have been recognized from the McMurdo Sound Region (Esposito et al., 2008), with most described species belonging to genera *Luticola, Hantzschia, Humidophila*, and *Muelleria* (Figure 6). Interestingly, centric/planktonic diatoms are effectively absent here (e.g., *Discostella, Chaetoceros, Stephanodiscus*, etc.), as are many freshwater genera that are ubiquitous elsewhere, including in other Antarctic regions (e.g., *Eunotia, Gomphonema, Neidium*, etc.). Since most initial work on MDV diatoms focused on lakes and ponds, little was known about Antarctic stream diatoms by the end of the 20th century. In fact, Jones (1996, p. 1439) wrote, “In contrast to work conducted in standing water bodies, there is very little information available on the diatom communities in running waters, although it is likely that diatoms are found in melt streams throughout the Antarctic.”

Since that time, we have learned much about MDV stream diatoms, especially with regard to their potential as indicator or sentinel taxa. Specifically, initial observations of diatoms living in the stream mats revealed that their community composition was comparable among the same mat types collected at the same time in a given reach of the same stream. This came in stark contrast to the high degree of reach‐scale patchiness observed in the cyanobacterial communities composing orange mats, for example (Alger, 1997; McKnight et al., 1998). Furthermore, it was observed that diatom relative abundances within these communities were responsive to interannual changes to the summer flow regime (e.g., Esposito et al., 2006), presumably due to species‐specific environmental preferences. Coupled with a relatively low number of species, these qualities have served as the primary motivation for incorporating diatom (over cyanobacterial) community composition into MDV stream monitoring.

Nonetheless, environmental selection is but one mechanism by which microbial communities are assembled, with the others being dispersal, diversification, and drift (Nemergut et al., 2013; Vellend, 2010). The first two of these mechanisms have been long assumed to be influential for MDV diatoms, as noted by West and West (1911, p. 265): “The comparative poorness of the Antarctic Alga‐flora may also be in part due to the greater severity of the climate combined with the remoteness of the Antarctic continent from other continental areas”—an interpretation that has not changed appreciably over the last century. However, the latter mechanisms, especially in combination with the others, have been less commonly considered. Thus, to illustrate how MDV diatoms make excellent models for studying community assembly in stream mats, we herein have discussed the four community assembly mechanisms as they apply to MDV diatoms, keeping in mind that these can easily apply to other MDV microorganisms as well.

### Selection

Selection is the most important community assembly mechanism from a biomonitoring perspective, since it assumes that environmental changes are reflected in community structure. Given the geographical setting, one of the most obvious challenges for MDV diatoms is the prolonged cold and dry conditions. Most MDV diatoms are classified as aerophilic (i.e., typical for soils) and have likely been selected for given their affinity for arid conditions (Esposito et al., 2008). Along with other MDV stream inhabitants, some genera are also psychro/cryophilic (cold‐loving), with polar strains of *Pinnularia borealis* being the most well‐known examples (Hejduková et al., 2019, 2024), although other MDV diatom genera likely share this character as well. Diatom community structure has been further linked to water temperature, with different species showing different thermal optima in culture (Darling et al., 2017), potentially helping to explain differences in diatom communities as a function of upstream/downstream position (McKnight et al., 1998; Wlostowski et al., 2019). However, it is difficult to disentangle the effect of longitudinal temperature changes from concomitant changes in flow and nutrients in practice, as explained below.

For organisms living in MDV GFSs, flow regime has been highly influential and is one of the characteristics most likely to change in the future. Accordingly, much work on MDV stream diatom communities has focused on their responses to flow (Esposito et al., 2006; Stanish et al., 2011, 2012; Stanish, O'Neill, et al., 2013). Researchers have observed that smaller, adnate diatoms, such as from the genera *Humidophila* and *Psammothidium*, have been more common in regularly flowing streams, whereas larger‐celled taxa (e.g. *Pinnularia borealis*) prefer drier conditions. Species from the genus *Luticola* are especially promising candidates for ecological indicators given their diversity (presently 12 recognized MDV species; Kohler, Kopalová, et al., 2015) and their relationships with flow regime. Specifically, gradients in streamflow intermittency correspond with changes in the identity and diversity of *Luticola* species present, and more intermittent streams host higher *Luticola* relative abundances (Stanish et al., 2012; Wlostowski et al., 2019). Finally, the small and lightly silicified *Fistulifera pelliculosa* may be among the most useful flow indicators, since it is, presumably, only lightly attached to the streambed sediments. As a result, it is readily mobilized during daily flow peaks (Stanish et al., 2024) and seems to accumulate in mats over the summer and following disturbances (Kohler, Chatfield, et al., 2015).

Different nutrient conditions may also favor different diatom species, with greater concentrations specifically favoring *Fistulifera pelliculosa* and within‐reach variability in diatom communities potentially corresponding with the presence (or absence) of hyporheic upwelling zones (Kohler et al., 2016). Dissolved Si may also have a role in deciding diatom community structure, given that it is a major component of diatom cell walls. For example, in shorter streams where Si concentrations are low (from limited weathering), common diatom taxa have been smaller and more lightly silicified than those inhabiting longer streams with greater Si concentrations. Although more work is necessary to determine if this link is causal, diatom uptake has been, nonetheless, seemingly sufficient to alter the stream Si isotopic pool (Hatton et al., 2020).

Lastly, the different mat types themselves may select for different taxa due to the different microconditions they promote, as observed on the Antarctic Peninsula (e.g. Kopalová et al., 2019), although only a paucity of MDV studies exist on the topic (e.g. Schulte et al., 2022). In one study, Stanish et al. (2024) compared black and orange mat diatom communities in Von Guerard Stream, as well as communities inhabiting sediments with no mats, and observed that *Hantzschia amphioxys* f. *muelleri* and *Navicula seibigiana* were more common in black mats; *Stauroneis latistauros*, *Craticula molestiformis*, and *Luticola laeta* in orange mats; and *Halamphora oligotrophenta*, *Fistulifera pelliculosa*, and *Mayamaea atomus* in bare sediments. Although difficult to generalize, these results suggest that preferences do occur, and we hypothesize that more desiccation‐tolerant taxa should inhabit black mats, flow‐resistant taxa should inhabit orange mats, and the live‐quick‐die‐young diatoms inhabit green mats and sediments.

### Dispersal

Understanding organismal dispersal within and among habitats is a basic necessity for understanding their distributions. Although all MDV diatoms are equipped with a raphe system (i.e., the slit in silica cell walls used for motility), raphe‐facilitated movement has been restricted to small spatial scales, such as vertical and lateral migration within mats (although the behavior of diatoms within their mat habitats is poorly studied as well). Contrary to active dispersal, passive diatom dispersal is likely much more effective in transporting organisms and in streams is largely unidirectional. Expanding upon the clockwise hysteresis pattern for transported mat biomass documented by Cullis et al. (2014), Stanish et al. (2024) showed the exact same pattern for diatom frustules and demonstrated that, although bare sediments contributed diatoms at all time periods, orange and black mats contributed the greater proportions of diatoms to the particulate organic matter pool over higher discharge periods both within and across summers. Thus, the mat type of origin, the time of day, and the magnitude of summer melt are all relevant for downstream diatom dispersal. This downstream flux of organisms may even promote the longitudinal structuring of diatom communities and could potentially outweigh the importance of selection in some cases (i.e., “mass effects”; Leibold et al., 2004), such as in the “moat” habitat surrounding the permanently frozen lakes (Stone et al., 2024).

Although downstream dispersal is essentially assured, the efficiency of among‐stream dispersal is less certain, although modeling exercises have indicated that some sort of intersite dispersal is necessary to maintain the MDV diatom metacommunity (Sokol et al., 2020). Given the lack of common organismal vectors (e.g., birds, mammals, fish), the primary dispersal agent is likely to be the wind. The MDVs are one of the windiest places on Earth, and numerous studies have suggested that the downvalley foehn winds (Nylen et al., 2004; Šabacká et al., 2012; Speirs et al., 2010) are important for organismal dispersal (Broady, 1996; Nkem et al., 2006; Wharton Jr et al., 1983). At the same time, upvalley “easterly” winds have been common in summer and have the capacity to bring with them some of the diverse taxa from the coast. Interestingly, Kohler et al. (2021) previously observed pond diatom communities inhabiting the “Stranded Moraines” to be intermediate between those from Ross Island and the MDV. Meanwhile, microbial diversity within Taylor Valley has been shown to increase toward the Ross Sea coast (Michaud et al., 2012; Stanish, Bagshaw, et al., 2013), and among streams, some of the highest diatom species richness values have been recorded from Wales Stream near New Harbor (Kohler, unpublished data), whereas some of the lowest were observed in Wormherder Creek near Taylor Glacier (Stanish et al., 2012; Wlostowski et al., 2019). Whether these patterns are primarily the result of “easterlies” (delivering diverse coastal taxa to the MDV), “westerlies” (promoting the accumulation of taxa toward the coast and discouraging upvalley dispersal), or neither remains uncertain.

Although dispersal by wind has been widely assumed, observing diatoms in the act of being transported is difficult indeed. Although Diaz et al. (2018) were among the first to show diatom valves from wind‐dispersed aeolian material in the MDV, the topic has only been explicitly studied by Schulte et al. (2022). In this latter work, the authors recovered living diatoms (verified through intact protoplasts and RNA) from aeolian collectors, providing strong evidence for wind dispersal of viable diatom cells. Furthermore, they presented evidence that MDV diatom dispersal was probably greatest within lake basins, low to moderate within valleys, and low among valleys. Smaller diatoms were also disproportionately observed in aeolian traps, helping to explain why most MDV diatoms (particularly the widespread ones) have been relatively small. Although this research has been an important first step, it remains to be investigated how the importance of dispersal compares with selection, among other mechanisms, in forming stream diatom communities within the MDV, as well as across the streams observed along Antarctica's other ice‐free coastal oases.

McMudro Dry Valley diatom genera are not the only aerophillic/psychrophilic genera on Earth—why are they here, and not others? Similarly, why aren't MDV diatoms observed anywhere else? Antarctica is isolated from other land masses by the Southern Ocean, and Antarctic ice‐free areas are infrequent and scattered, making organismal dispersal difficult—indeed, this scenario has also limited the dispersal of humans. Evidence that dispersal may be limiting in the Ross Sea sector was visualized in Sakaeva et al. (2016), who observed pond diatom communities between Ross Island and the MDV to be very different, despite similar environmental conditions separated by >60 km. Logically, it should only become harder to disperse over distances greater than this, such as at the continental scale. Biogeographically, Antarctica has been divided into at least 16 distinct regions based on its inhabitants (Terauds & Lee, 2016), although it is not clear if stream diatom communities reflect these units. In a study looking at Antarctic pond diatoms, communities were observed to strongly differ between sub‐, Maritime, and continental Antarctica and roughly corresponded with the 16 bioregions, all the while substantially simplifying with increasing latitude (Verleyen et al., 2021). Although the exact processes leading to this spatial structure are not entirely understood, they almost certainly involve dispersal limitation and subsequent diversification.

### Diversification

Although the importance of diversification is generally small when considering “normal” timescales for most ecological applications, it is vitally important for understanding the regional species pool, species distributions, and biogeography. Yet, we take much of this knowledge for granted, and although it is clear to us presently that MDV stream diatoms are globally unique, even just a few decades ago this understanding was not the case. For example, Seaburg et al. (1979, p. 73) stated, “A major question yet unanswered concerns the explanation for the occurrence of the particular algal floras in these areas which are so similar to those of other less remote regions on earth. That is, why is there no significant species or genus endemism in Antarctic oases?” Later, Jones (1996, p. 1444) stated for the diatoms, “Overall, there appears to be a rather low proportion of endemic taxa; however, the adoption of a finer species concept may well reveal a greater number of endemic species, but until sufficient studies have been made using a more reliable taxonomy it will not be clear how many species are endemic and how many are truly cosmopolitan.” In the intervening decades, diatom species boundaries, and our methods for determining them, have shifted markedly with the development of a finer grained taxonomy. Contrary to a century ago, it is more likely now that we would assume that a given MDV diatom is endemic rather than cosmopolitan, unless proven otherwise.

Diatom communities recovered from MDV lake sediments spanning the last several thousand years look essentially identical to the modern ones from corresponding habitats (Kellogg et al., 1980; Konfirst et al., 2011; Spaulding et al., 1997; Whittaker et al., 2008), indicating relative metacommunity stability at least over this timescale. However, this is not the case when looking at diatom communities from some millions of years ago, as almost nothing is the same. Intriguingly, in their analysis of MDV samples deposited during the Miocene, Pinseel et al. (2021) observed that genera that were at the time currently diverse and abundant in the MDV, such as *Luticola* and *Hantzschia*, were missing in previous times, whereas genera absent from the MDV in 2021, such as *Gomphonema, Eunotia*, and *Neidium*, had been present previously. The MDV prior to the Miocene were likely much warmer, and therefore, the diatoms composing the flora during these times probably required more stable aquatic habitats for their persistence. Thus, much of the original MDV diatom diversity went extinct or was extirpated in the intervening years with the formation of the ice sheets, while some presumably found refugia in nearby oases (Stevens & Mackintosh, 2023). These “survivors” and “colonists” underwent subsequent diversification, which almost certainly resulted in the high level of endemism visible today. For example, in the genus *Luticola*, of the 12 currently accepted species present in the McMurdo Sound Region, eight have only been known from the region and are putatively endemic to it (Kohler, Kopalová, et al., 2015).

Collectively, this history potentially makes MDV diatoms a microbial version of the “species flocks” observed in the East African Great Lakes and the Galapagos Islands (Kociolek et al., 2017). Coupled with a harsh environment and dispersal limitation, the combination of mechanisms has almost certainly been responsible for the manifestation of the different bioregions discussed in the previous section. Yet, virtually everything we know about Antarctic diatoms at present is reliant on the use of a morphological species concept, with species identifications performed solely via microscopy. Thanks to recent research, we now know that diatoms are more diverse than their appearances suggest, since diatoms with the same appearance can also have different genomes—a situation known as “cryptic diversity.” Such diversity has been famously demonstrated in the case of *Hantzschia amphioxys* (Souffreau et al., 2013) and *Pinnularia borealis* (Pinseel et al., 2020), both of which are present in the MDV and were historically assumed to have cosmopolitan distributions. As a result, diatom diversity is underestimated, and endemicity (and therefore regional patterns) may be much more pronounced than currently appreciated. Altogether, given its geological history, relatively low diversity, and simple environment, the MDV (and Antarctica in general) represent an ideal place to study controls on, and patterns in, diatom evolution.

### Drift

Although ecological drift is likely to be an important component of diatom community assembly, it is also the most difficult to quantify and, therefore, has been the least frequently addressed. When drift has been addressed, it has been generally inferred from unexplained variation rather than directly quantified. Yet, controlled laboratory study has shown that it can produce non‐trivial variances in microbial communities (Fodelianakis et al., 2021), and new phylogenetic approaches (e.g., Ning et al., 2020) offer alternative ways to quantitatively infer drift as a community‐structuring mechanism, including for diatoms (e.g., Keck et al., 2016; Keck & Kahlert, 2019). Recently, Rimet et al. (2025) showed that drift was the most important ecological process shaping the diatom communities of high‐alpine lakes. Although the reason is not entirely clear, Nemergut et al. (2013) suggested drift to be most influential when selective pressures are weak and diversity is low. We speculate that in MDV streams, relatively low numbers of dispersing cells (most of which are similarly adapted to the harsh conditions of Antarctica), coupled with a paucity of suitable habitat, may greatly reward the first colonizers with a numerical advantage over those that may come later—a phenomenon known to ecology as “priority effects” or “monopolization” (De Meester et al., 2016)—even if the specific identity of the first successfully colonizing cells was completely random. Indeed, it may not come as a surprise that species living within the thick overwintering mats would find preemptive competitive success over potential colonists arriving with onset of flow in summer. Therefore, drift may represent an important process for MDV stream diatom communities and may be particularly influential during the primary succession of entirely new systems expected to emerge as the region warms (Gooseff et al., 2011; Lyons et al., 2005; Nielsen et al., 2012).

## BIOLOGICAL RESPONSES TO CLIMATE CHANGE

**FIGURE 7:**
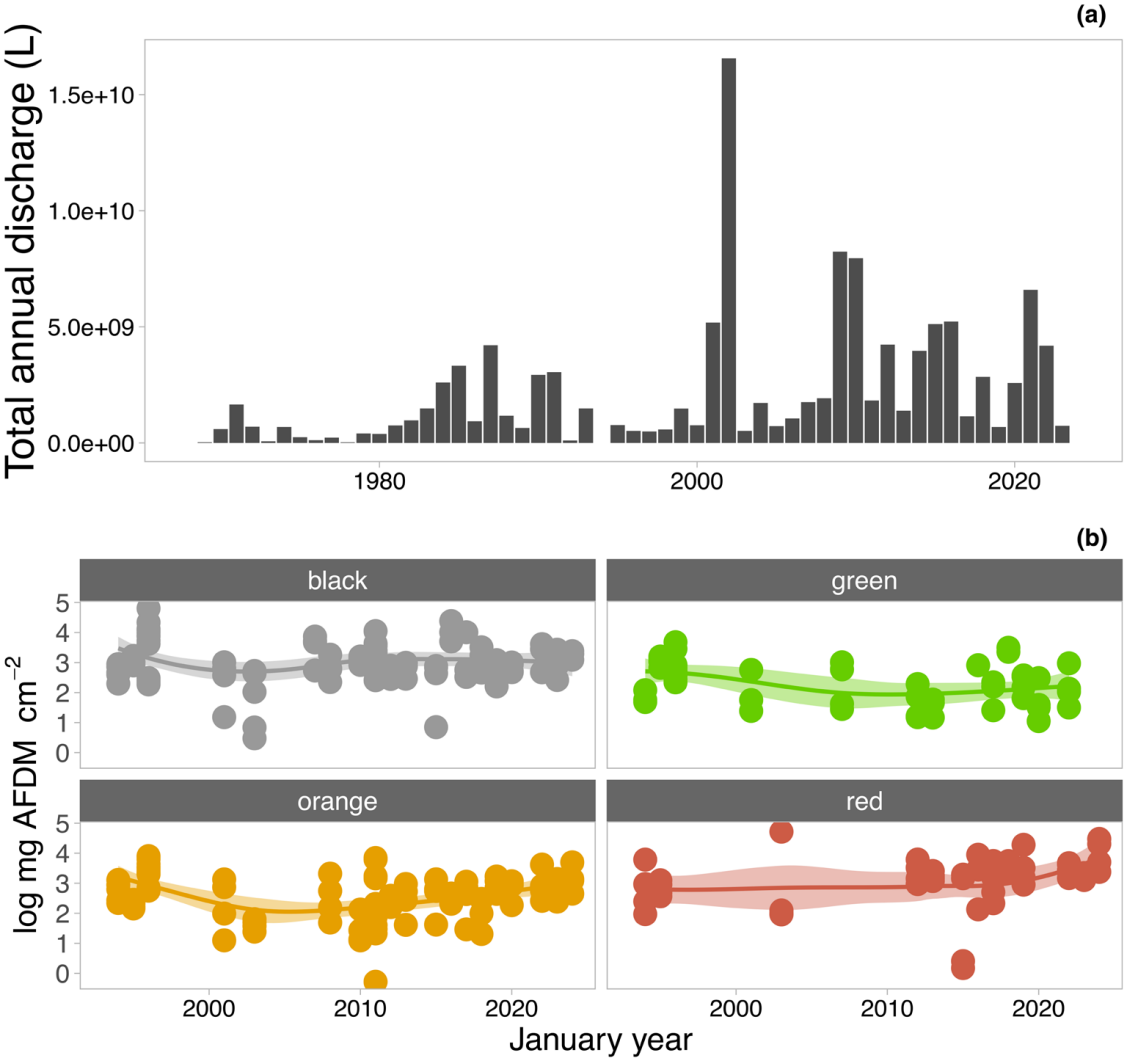
Top (a) panel shows the total annual discharge for the Onyx River at the Lake Vanda gaging station (January 1969–2023 summers), which has the longest running hydrological dataset of any MDV stream. Below (b), biomass (as ash‐free dry mass, AFDM) for Canada Stream black, green, orange, and red mats are plotted (January 1994–2024 summers). Hydrological data are from Gooseff and McKnight (2024) and mat biomass data from Zeglin and McKnight (2025).

Glaciers are shrinking worldwide and, in the process, altering the flow regimes of GFSs (Huss & Hock, 2018; Milner et al., 2017). Although these hydrological shifts certainly affect the physical and chemical template downstream, they will also undoubtedly alter the structure and function of GFS biota. Yet, it remains unclear *how* communities will respond, partially because long‐term monitoring is necessary to adequately capture the intra‐ and interannual hydrological variability needed to study biological feedback in intermittent streams. Thanks to ongoing monitoring efforts in the MDV GFSs, these systems provide an excellent opportunity to study how stream biota might respond to a changing flow regime. As discussed previously, MDV GFSs hydrographs are highly variable, yet discharge monitoring initiated in the late 1960s for the Onyx River has revealed some broad patterns over the last half‐century (Figure 7a). Specifically, annual discharges measured during the 1970s were relatively low, but they increased during the 1980s, leading to the rise in Lake Vanda's shoreline that prompted the abandonment of Vanda Camp. Annual discharges were again relatively low during the 1990s, coinciding with the first decade of monitoring by the MCMLTER, and this has often been referred to as the “cool period” (Doran et al., 2002; Obryk et al., 2020). However, this trend was interrupted during the aptly named “flood year” of 2002 (Doran et al., 2008), and flows have remained high but variable up to the present.

To investigate how these broad patterns corresponded with the biomasses of different mat types, Gooseff et al. (2017) utilized a 20‐year MCMLTER dataset consisting of samples taken at several monitored stream transects within Taylor Valley. They observed both orange and black mat biomass decreased during the cool period up until the flood year (Figure 7b). Then, in the high‐but‐variable summers that followed, black mats were quick to regain their initial biomass, while orange mat biomass lagged behind. One hypothesis for these diverging patterns is that moderately high and consistent flows may serve to stimulate black mat growth by better hydrating marginal habitats, whereas the same moderate flows may be comparatively less beneficial (or even counterproductive) to the already submerged orange mats. Functional differences have also been hypothesized to be a mechanism: Given N limitation in the Fryxell Basin (where most observations were derived), one possibility is that orange mat recovery rates may be reliant upon the regrowth of N‐fixing black mats to first rebalance N budgets (Kohler et al., 2023). Complicating generalizations is the geomorphological diversity of MDV streams. Analyzing the same 20‐year dataset, Kohler, Stanish, et al. (2015) showed that trends in mat biomass have differed depending if the biomass came from low‐ or high‐coverage streams. For example, in low‐coverage streams, orange mat biomass was positively associated with high flows, whereas orange mat biomass in high‐coverage streams was negatively associated with high flows. Kohler, Stanish, et al. (2015) speculated this observation is because in low‐coverage streams, greater discharge may lead to greater biomass since it opens more stable side channels for habitation (e.g., Vincent & Howard‐Williams, 1986), whereas it only serves to scour biomass in high‐coverage streams, given there is initially more to lose.

Therefore, in terms of how MDV mats will respond to changing hydrological conditions, results thus far have been, sometimes, counterintuitive, and there does not seem to be a one‐size‐fits‐all scenario. Instead, the outcome is likely stream and mat‐type specific, ultimately hinging upon that fine line of having enough flow to grow and reproduce but not so much that biomass is scoured from the substrate. In addition, hydrology represents only one of several possible gain/loss processes, and any future increases to MDV stream mat biomass may be offset by increases in other pathways (e.g., decomposition, wind). Another outstanding question is how these reach‐scale observations will be reflected in the MDV as a whole. Although MCMLTER stream transects were chosen to be representative of the entire stream length, MDV streams, like streams worldwide, are remarkably heterogeneous and vary considerably over their full longitudinal extent. Thus, the challenge for future researchers will be to account for these different loss and gain processes and apply them to the regional scale.

A separate question is what changing conditions may mean for local diversity. Using diatoms as a model, we know that the presence/absence of species has remained relatively stable within respective habitats over time (Konfirst et al., 2011; Spaulding et al., 1997). Furthermore, although diatom extinctions have taken place in Antarctica in the past (Pinseel et al., 2021), the last major extinction event presumably took place because the environment got too cold. Since most MDV microbes are regularly operating beneath their optimal temperatures, it is possible that warmer temperatures (and associated hydrological changes) will not inevitably lead to species extirpations/extinctions but rather primarily alter the structure of existing communities. As an example, Stanish et al. (2011) observed that the relative abundances of several diatom species from Green Creek were shuffled following the 2002 flood year, although all species remained accounted for, and no other streams exhibited comparable shifts. That analysis, however, only considered orange mats, and it is possible that black mat diatom communities (for example) are more sensitive to changing conditions given their position at stream margins. Furthermore, warmer summers with more regular flow conditions might eventually favor “genuinely” cosmopolitan diatom taxa, whereas endemic taxa may be favored under hydrological conditions observed during colder summers (Esposito et al., 2006). Although warmer conditions may also facilitate alien (i.e., non‐Antarctic) taxa to settle here, ultimately any new colonists successful in dispersing to the MDV must also survive the 24‐h darkness of polar winters and the associated freezing and desiccation to permanently establish—for all of which, the current community already has the upper hand. At the same time, the introduction of taxa from one Antarctic bioregion to another (e.g., the Antarctic Peninsula) is a very serious threat, and one that is too infrequently discussed.

Importantly, in order to assess future changes to MDV GFS diversity, it is first necessary to know what lives there. As discussed above, we are only beginning to quantify MDV microbial diversity, despite the fact that microbes are the dominant form of life there. Given that range‐restricted taxa have been observed to make up more than half of the bacterial sequence diversity in GFSs globally (Ezzat et al., 2025), we suspect that the level of microbial uniqueness in MDV GFSs is very high, and comparisons with benthic communities from other GFSs worldwide are necessary to investigate how these systems compare with the rest of the world. Yet we should do so soon—given both a changing climate (which alters the environmental template) and a growing number of humans accessing the continent (serving as a dispersal vector), there is a real danger that Antarctica's microbiome will become irreversibly homogenized (Hughes et al., 2015, 2019), creating an urgent need to study and preserve the samples already in our possession (Hawes et al., 2025). In addition to establishing baseline conditions, archived samples also ensure science can continue despite rising costs and complications associated with changing government policies and infrastructure while at the same time minimizing the ecological footprints that come with further sampling (O'Brien et al., 2022). Finally, in anticipating future change, we must make sure that current diversity protection mechanisms match our goals (Hawes et al., 2025). With ~6% of MDV stream area currently protected under Antarctic specially protected areas (Wright, Brooks, et al., 2024) and with a rapidly changing world on the horizon, the time is right to inspect our shared values and desired outcomes when it comes to Antarctic diversity.

## SYNTHESIS AND IMPLICATIONS

Rather than being the “valleys of the dead” Scott described, the last half century has revealed that microbial life abounds in the MDVs, not least of all in the GFSs. Although early efforts during Antarctica's Heroic Age were primarily focused on discovery, most recent efforts in the MDV have been dedicated to detecting environmental change. And rightly so—the MDV GFSs are excellent systems with which to study climate change feedback given their direct link with glacier melt, with the stream mats on the front lines. In this review, we described three color‐defined categories of stream mats, which are composed of microorganism groups shared with other stream systems globally. We further suggested that these MDV GFS mats are structured by a unique combination of habitat characteristics, with their presence, biomass, and coverage primarily determined by geomorphology, suspended sediment load, and hydrological conditions, among other factors. The high tolerance of the MDV mats to adverse conditions (e.g., freezing, desiccation, UV radiation), combined with the long‐term conditioning of the hyporheic zone by microbial biomass, interact to sustain these stream ecosystems over long timescales, and we suggest that these processes are likely important in intermittent stream ecosystems elsewhere. At a finer scale, much remains to be learned about the diversity of MDV mat residents, the presence of which are almost certainly the products of physical isolation, adaptation to (and selection by) harsh environmental filters, and subsequent speciation. Collectively, we argue that the MDV GFSs are excellent model ecosystems for studying global change microbiology, and the mats and their diatoms make ideal model groups for biological monitoring to understand processes that determine the resilience of microbial communities inhabiting stream ecosystems. Yet, given their differences, we also argue that mats and diatoms are best suited to answer *different* questions, complementing each other in helping us understand Antarctic stream ecology.

## FUTURE CONSIDERATIONS

### Diversity

There is a pressing need to document MDV GFS diversity. Fungi, protists, bacteria, archaea, and viruses remain minimally explored because the best techniques to study them have not yet been applied. The same is true for the most studied group—the diatoms—given that the current methods for researching them have almost not changed since the Heroic Age (Fritsch, 1912, 1917; West & West, 1911). Yet, today we know that morphology alone is not sufficient to fully characterize diatom diversity because of phenotypic plasticity (i.e., the same species exhibiting different morphologies) and cryptic diversity (i.e., different species being morphologically indistinguishable). Although sounding basic, cataloging diversity is absolutely critical for assessing evolutionary drivers, biogeographical patterns, and the outcomes of future change.

### Functionality

There is a glaring lack of information about how MDV stream microbial diversity translates into stream ecosystem functioning. Although photosynthesizers have well‐appreciated functions, MDV mats harbor a vast diversity of organisms working across redox gradients and utilizing much less‐appreciated metabolic pathways (e.g., Vigneron et al., 2018). While “omics” approaches remain limited in MDV stream research, they are necessary to fully investigate what is metabolically possible and where within GFSs (e.g., Michoud et al., 2025) and may ultimately help to answer some of the longest held questions about mats here and elsewhere (e.g., how can mats be so abundant when nutrient concentrations are so low?). On top of functional diversity, it is also necessary to learn what influences functional process rates. For example, how will mats respond to changes in temperature, discharge, and radiation in terms of nutrient (e.g., N‐fixation, denitrification) and carbon (respiration, production) cycling?

### Landscape‐level change

Characterizing heterogeneity in MDV stream mat biomass and coverage beyond the reach scale remains a challenge. Augmenting current methodologies with remote sensing and LiDAR is potentially game changing for monitoring the distribution of mat biomass in the future (Power et al., 2020, 2024), allowing investigators to identify large‐scale changes (both spatial and temporal) and drivers (such as thermokarst slumping and streambed erosion; e.g., Gooseff et al., 2016; Levy et al., 2013, 2018). Having a grasp of these large‐scale processes can help to give an idea of other changes expected across the landscape. For example, constraining the expansion/contraction in the biomass of individual mat types over entire streams will help answer questions about what these changes might also mean for MDV diversity and biogeochemistry.

### Antarctica in context

Future work should strive to link with other research programs, laboratories, and disciplines focused not only on Antarctica but also on systems worldwide. Comparisons of MDV mats with those from other GFSs, as well as benthic microbial consortia observed in other environments (e.g., hypersaline, hot springs, deserts), would give MDV stream mats context, and involving other disciplines could lead to completely different research questions. Furthermore, understanding how MDV stream biota diversifies, how communities are assembled, and how biogeographical patterns manifest across Antarctica will almost certainly help provide an understanding of similar phenomena in the comparatively more complicated systems of lower latitudes. Importantly, the results may also suggest the trajectory of future microbial ecosystems following severe climate change and extinction events.

### Societal considerations

The MDV are changing, with hydrological shifts (Gooseff et al., 2017; Kohler, Stanish, et al., 2015) and unprecedented weather anomalies (Barrett et al., 2024; Doran et al., 2008) possibly foreshadowing new conditions to come. At the same time, given global trends and pressures, it is conceivable that the future will also see greater activity from scientists, tourists, and commercial interests in Antarctica. The combination of these presents an elevated risk of invasive species establishment, pollution, and physical disturbance. However, it remains an open question how such developments will affect MDV diversity and, in turn, how well current management plans accommodate diversity preservation and management goals (Wright, Brooks, et al., 2024). In this context and in addition to a thorough census of MDV life, a physical, accessible biorepository with specimens and nucleic acids should be a priority.

## AUTHOR CONTRIBUTIONS

**Tyler J. Kohler:** Conceptualization (lead); data curation (lead); formal analysis (lead); funding acquisition (supporting); investigation (lead); visualization (lead); writing – original draft (lead). **Ian Hawes:** Investigation (supporting); validation (supporting); writing – review and editing (supporting). **Adrian Howkins:** Investigation (supporting); validation (supporting); writing – review and editing (supporting). **Lydia H. Zeglin:** Conceptualization (supporting); data curation (supporting); funding acquisition (supporting). **Mike N. Gooseff:** Conceptualization (supporting); data curation (supporting); funding acquisition (supporting). **Diane M. McKnight:** Conceptualization (supporting); investigation (supporting); validation (supporting); writing – review and editing (supporting).
